# Tradução para a língua portuguesa do artigo original em inglês “ *The International Continence Society* (ICS) *report on the terminology for adult male lower urinary tract and pelvic floor symptoms and dysfunction”*

**DOI:** 10.31744/einstein_journal/2021AE5694

**Published:** 2021-07-01

**Authors:** Carlos Arturo Levi D’Ancona, Ricardo Luís Vita Nunes, Alberto Azoubel Antunes, Rogerio de Fraga, Alcides Mosconi, Luis Abranches-Monteiro, Bernard Haylen

**Affiliations:** 1 Universidade Estadual de Campinas CampinasSP Brasil Universidade Estadual de Campinas , Campinas , SP , Brasil .; 2 Hospital Militar de Área de São Paulo São PauloSP Brasil Hospital Militar de Área de São Paulo , São Paulo , SP , Brasil .; 3 Universidade de São Paulo São PauloSP Brasil Universidade de São Paulo , São Paulo , SP , Brasil .; 4 Complexo Hospital de Clínicas Universidade Federal do Paraná CuritibaPR Brasil Complexo Hospital de Clínicas , Universidade Federal do Paraná , Curitiba , PR , Brasil .; 5 Hospital da Saúde do Homem São PauloSP Brasil Hospital da Saúde do Homem , São Paulo , SP , Brasil .; 6 Hospital Beatriz Ângelo Lisboa Portugal Hospital Beatriz Ângelo , Lisboa , Portugal .; 7 University of New South Wales Sydney Australia University of New South Wales , Sydney , Australia .

**Keywords:** Disfunção do trato urinário inferior, Sintomas do trato urinário inferior, Masculino, Imagem do trato urinário masculino, Urodinâmica masculina, Terminologia

## Abstract

**Introdução:**

No desenvolvimento da terminologia do trato urinário inferior, devido à sua crescente complexidade, a terminologia para os sintomas e a disfunção do trato urinário inferior masculino e assoalho pélvico precisa ser atualizada, utilizando abordagem específica masculina e por meio de um relatório de consenso clinicamente embasado.

**Métodos:**

Este relatório combina a contribuição de membros do *Standardisation Committee* da *International Continence Society* em um Grupo de Trabalho com especialistas reconhecidos no campo, assistido por muitos julgadores externos. Categorias clínicas centrais apropriadas e uma subclassificação foram desenvolvidas para fornecer uma codificação numérica para cada definição. Um extenso processo de 22 rodadas de revisão interna e externa foi desenvolvido para examinar exaustivamente cada definição, com tomada de decisão por opinião coletiva (consenso).

**Resultados:**

Um relatório de terminologia para os sintomas e a disfunção do trato urinário inferior e do assoalho pélvico foi desenvolvido, abrangendo cerca de 390 definições/descritores separados. É clinicamente embasado nos diagnósticos mais comuns definidos. A clareza e a facilidade de uso foram os principais objetivos para torná-lo interpretável por profissionais e estagiários em todos os diferentes grupos de especialidades envolvidos na disfunção do trato urinário inferior e do assoalho pélvico masculino. Imagens específicas para homens (ultrassonografia, radiologia, tomografia computadorizada e ressonância magnética) foram um adicional importante, enquanto figuras apropriadas foram incluídas para complementar e ajudar a esclarecer o texto.

**Conclusões:**

Um relatório de terminologia com base em consenso para sintomas e disfunção do trato urinário inferior e do assoalho pélvico masculino foi produzido visando ser um auxílio significativo para a prática clínica e um estímulo para a pesquisa.

## INTRODUÇÃO

Atualmente, não há um único documento abordando todos os elementos necessários para os diagnósticos aplicáveis à disfunção do trato urinário inferior (TUI) e do assoalho pélvico (AP) em adultos (totalmente crescidos e fisicamente maduros ***novo*** ). ^( 1 )^ De fato, as próprias entidades diagnósticas podem não ter sido completamente definidas. O termo “diagnóstico” é definido como “a determinação da natureza de uma doença; **clínica:** feita a partir do estudo dos sintomas e sinais de uma doença; ^( 1 )^ “ **laboratorial:** ” opções de investigação a serem mencionadas. Tal relatório específico exigiria uma descrição detalhada da terminologia para todos os sintomas, os sinais e as investigações urodinâmicas para a disfunção do TUI e do AP masculinos, das imagens associadas a essas investigações e dos diagnósticos mais comuns.

Pode ter sido possível, no passado, combinar toda a terminologia da função do TUI para homens, mulheres e crianças em um único relatório. A *International Continence Society* (ICS) liderou a terminologia para disfunção do TUI ao longo de décadas, empregando relatórios combinados ou genéricos. Os relatórios de 1988 ^( 2 )^ e 2002 ^( 3 )^ do Comitê de Padronização de Terminologia são exemplos disso. Com a crescente especificidade e complexidade dos diagnósticos em ambos os sexos, relatórios combinados, sem falar na tentativa de cobrir “todos os grupos de pacientes de crianças para idosos”, ^( 3 )^ podem agora ser um anacronismo. Com a evidência de que a ausência de diagnósticos femininos específicos, bem como outras terminologias específicas femininas, pode não ter sido favorecida por uma abordagem combinada, ^( 4 )^ foi desenvolvido e publicado um relatório conjunto da *International Urogynecological Association* (IUGA) e da ICS sobre a terminologia sobre a disfunção do AP feminino. ^( 5 )^ O relatório de 2002 ^( 3 )^ ainda proveu a terminologia masculina tradicional e algumas modificações úteis – muitas das quais são repetidas neste documento. O relatório atual, com o grande número de definições ***novas*** e ***modificadas*** , reconhece que uma atualização específica masculina da terminologia para sintomas e disfunção de TUI e AP é agora oportuna.

Espera-se que algumas das vantagens observadas no documento específico sobre AP feminino ^( 5 )^ possam ser vistas aqui neste documento sobre AP masculino, como cobertura (i) mais abrangente da terminologia masculina específica; (ii) maior coerência e facilidade de uso; (iii) maior especificidade dos diagnósticos masculinos e (iv) comunicação mais precisa para fins clínicos e de pesquisa. É também um objetivo deste documento desenvolver uma terminologia masculina geral, formando uma terminologia “espinha dorsal” ou “central”, para facilitar a atualização de outras subcategorias de terminologias específicas para homens. Existiram outros sete documentos terminológicos femininos fragmentados relacionados ao AP (IUGA/ICS), ^( 6 - 12 )^ tendo sido todos publicados após a produção do documento conjunto inicial da IUGA/ICS sobre disfunção do AP feminino. ^( 5 )^ Os autores desse documento ^( 5 )^ gentilmente permitiram que o modelo desse relatório fosse usado como base para o relatório atual. Quatro outros relatórios de terminologia masculina foram iniciados sobre (i) disfunção anorretal masculina; (ii) tratamento cirúrgico da disfunção do TUI masculino; (iii) saúde sexual em homens com disfunção do TUI/AP e (iv) manejo conservador da disfunção masculina de TUI/AP, para suceder a publicação deste relatório “central”.

Este relatório de terminologia é inerente e apropriadamente um documento de definição, o qual reúne as definições desses termos, isto é, palavras usadas para expressar um conceito definido em um ramo específico de estudo ^( 1 )^ – neste caso, a terminologia masculina central. A ênfase tem sido abrangente e inclui os termos em uso corrente na literatura pertinente revisada por pares. O objetivo é auxiliar a prática clínica e a pesquisa. Notas explicativas sobre definições foram referidas, sempre que possível, na seção notas de rodapé (indicada por “ _NR_ ”). A tabela 1 lista o número de definições (i) novas e (ii) modificadas e o (iii) total por seção, comparadas com os anteriores relatórios inclusivos de homens. ^( 2 , 3 )^

**Tabela 1:** Definições novas e modificadas (comparadas com relatórios anteriores inclusive para homens (2,3)

Seção	Definições/descritores novos	Definições/descritores modificados	Total
Introdução & sintomas	60	23	118
Sinais	73	14	98
Investigações	23	25	106
Imagem	42	N/A	42
Diagnósticos	14	9	26
Total	211 (54%)	71 (18%)	390

Tal como acontece com seu equivalente de terminologia feminina, as qualidades para um relatório de terminologia específica do sexo masculino devem ser:

1. Fácil de usar: deve ser entendido por todos usuários clínicos e de pesquisa.
2. Com base na clínica nos sintomas, sinais, investigações validadas e exames de imagem devem ser apresentados para uso na elaboração de diagnósticos. As seções 1 a 4 abordam os sintomas, os sinais, as investigações urodinâmicas e as modalidades atuais de imagem associadas, usadas rotineiramente no consultório, no laboratório de urodinâmica ou no departamento de imagens para fazer esses diagnósticos. Não se considera que os leitores estejam limitados a especialistas médicos, o que representa um exame físico básico mais extenso (vide seção 2). Investigações radiológicas relacionadas, tomografia computadorizada (TC) e ressonância magnética (RM), bem como descrição da eletromiografia (EMG), foram incluídas. Este relatório limita a terminologia para a disfunção neurogênica do tratro urinário inferior (DNTI), uma vez que isso é coberto por um relatório separado da ICS. ^( 13 )^
3. A seção 5 abrange diagnósticos mais comuns de disfunção do TUI e do AP masculinos. Os termos ^( 3 )^ “observação urodinâmica” e “condição” (não médica) não foram utilizados neste relatório. O escopo do relatório excluirá (i) patologia diagnóstica (sangue, urina e histologia); (ii) investigações mais invasivas que exigem anestesia (iii)e tratamentos com base em evidências para cada diagnóstico.
4. Origem: quando a definição existente de um termo (de uma das múltiplas fontes utilizadas) for considerada apropriada, essa definição será incluída e devidamente referenciada. Muitos termos na função do TUI e do AP masculinos, devido ao seu uso a longo prazo, tornaram-se agora genéricos, como é evidente por sua listagem em dicionários médicos.
5. Capaz de fornecer explicações: quando uma explicação específica for considerada apropriada para explicar uma alteração de definições anteriores ou para qualificar a definição atual, ela será incluída como um adendo a este documento ( *NR 1, 2, 3* ...). Sempre que possível, os princípios médicos com base em evidências serão seguidos.

Como em relatórios anteriores do ICS, ^( 2 , 3 , 5 )^ quando uma referência é feita para todo o órgão anatômico, a *bexiga urinária* , o termo correto é bexiga. Quando a estrutura muscular lisa, conhecida como *músculo detrusor vesical* , está sendo discutido, então o termo correto é “detrusor”. Sugere-se que o reconhecimento desses padrões em publicações escritas relacionadas a sintomas e à disfunção do TUI e do AP masculinos seja indicado por uma nota de rodapé na seção métodos e materiais ou seu equivalente, como segue: “métodos, definições e unidades estão em conformidade com os padrões recomendados pela ICS, exceto onde especificamente indicado”.

## SEÇÃO 1: SINTOMAS (ARMAZENAMENTO, ANULANDO, PÓS-ANULAÇÃO)

**Sintoma:** qualquer estado que determine a alteração de sensações, funções ou estruturas normais é uma indicação de um problema de saúde ou doença. Os sintomas podem ser informados voluntariamente ou perguntados ao paciente ou, também, descritos pelo acompanhante do indivíduo ou cuidador. ^( 2 , 3 , 5 )^

**Queixa:** a descrição do sintoma. ^( 1 )^ **( *Novo* )**

**Principal queixa:** o sintoma que o paciente declara como a principal queixa para ele procurar atendimento médico. ^( 1 )^ **( *Novo* )**

O grau de “incômodo (preocupação)” para outros sintomas pode ser variável. ^( 14 )^ **( *Novo* )**

**Sintoma do trato urinário inferior (STUI):** sintoma relacionado ao TUI. Pode estar relacionado com a bexiga, a próstata, a uretra e/ou o AP adjacente ou órgãos pélvicos, ou, às vezes, referir-se à inervação anatômica similar, como, por exemplo, o ureter distal. **( *Novo* )**

## SINTOMAS DE ARMAZENAMENTO

**1.1** **Sintomas de armazenamento:** sintomas do TUI ocorrendo durante a fase de armazenagem na bexiga. **(** ***Novo*** **)**

### Sintomas gerais de armazenamento

**1.1.1 Aumento da frequência urinária:** queixa de que a micção ocorre com mais frequência do que o considerado normal pelo indivíduo (ou cuidador). ^( 3 , 5 )^ A hora do dia e o número de esvaziamentos não são especificados. ( ***Novo*** **)**
**1.1.2 Aumento da frequência urinária durante o dia:** a queixa de que o esvaziamento é mais frequente durante o tempo de vigília – diferente daquele considerado normal pelo indivíduo (ou cuidador). ^( 3 , 5 )^_NR 1.1_ Nota Bene (NB): polaciúria. _NR 1.2_ ( ***Modificado*** **)**
**1.1.3 Noctúria:** o número de vezes que se urina durante o período principal do sono. Tendo acordado para urinar pela primeira vez, cada micção deve ser seguida pelo sono ou intenção de dormir. Isso deveria ser quantificado usando-se um diário miccional. ^(^^18^
**1.1.4 Poliúria (sintoma global):** queixa de que, durante o dia, o volume de eliminação da urina durante as 24 horas é notadamente maior do que em experiências anteriores. _NR 1.4_ **(** ***Novo*** **)**
  **1.1.4.1 Poliúria diurna:** a queixa é de que, no decorrer do dia, o volume eliminado de urina é notadamente maior do que em experiências anteriores. **(** ***Novo*** **)**
  **1.1.4.1 Poliúria noturna (sintoma):** queixa de urinar grandes volumes durante a noite. **(** ***Novo*** **)**

### Sintomas sensoriais

**1.1.5** **Sintomas (sensoriais) de enchimento da bexiga:** sensações anormais experimentadas durante o enchimento da bexiga. ^( 1 )^ **(** ***Novo*** **)**
  **1.1.5.1 Aumento da sensação de enchimento da bexiga:** queixa de que a sensação de enchimento da bexiga ocorre prematuramente e é mais intensa ou persistente do que antes. ^( 3 , 5 )^ **(** ***Modificado*** **) NB:** isso difere de urgência, pelo fato de que a micção pode ser postergada, apesar do desejo de esvaziamento.
  **1.1.5.2 Urgência:** queixa de um desejo obrigatório e repentino de urinar e que é difícil de adiar. ^( 3 , 5 )^_NR 1.5, NR 1.6_
  **1.1.5.3 Redução da sensação de bexiga cheia:** queixa de que a sensação de bexiga cheia é menos intensa ou ocorre mais tardiamente do que antes. **(** ***Modificado*** **)**
  **1.1.5.4** **Ausência de sensação de bexiga cheia:** queixa de ausência de sensação de bexiga cheia e ausência de um desejo miccional. ^( 3 )^
  **1.1.5.5 Sensação não específica (atípica) de enchimento da bexiga (disestesia da bexiga):** queixa de sensação de enchimento da bexiga anormal, como, por exemplo, a percepção de um vago inchaço abdominal, sintomas vegetativos (náusea, vômitos e fraqueza) ou espasticidade. **(** ***Modificado*** **)**_NR 1.7_ Isso difere de uma sensação normal de bexiga cheia ou dor, pressão ou desconforto da bexiga.

### Sintomas de incontinência

**1.1.6 Sintomas de incontinência urinária:** ^( 16 )^ perda involuntária de urina, que acontece durante a fase de armazenamento da bexiga. _NR 1.8, NR 1.9._ **(** ***Novo*** **)**
  **1.1.6.1 Incontinência urinária (sintoma):** queixa de perda involuntária de urina. ^( 3 , 5 )^_NR 1.9_
  **1.1.6.2** **Incontinência urinária de urgência:** ^( 3 , 5 )^ queixa de perda involuntária de urina associada com urgência.
  **1.1.6.3 Incontinência urinária por esforço:** queixa de perda involuntária de urina em esforços ou esforço físico, incluindo atividades esportivas ou no espirro, ou na tosse. ^( 3 , 5 )^ **NB**: O termo “ *incontinência relacionado* à *atividade* ( *esforço* )” deve ser usado em algumas línguas para evitar confusão com estresse psicológico. _NR 1.10_
  **1.1.6.4 Incontinência urinária mista:** queixa de ambos (esforço e urgência), ou seja, perda involuntária de urina associada com a urgência, assim como com o esforço ou esforço físico, que inclui atividades esportivas ou no espirro ou na tosse (esforço). ^( 3 , 5 )^
  **1.1.6.5 Enurese:** queixa de incontinência intermitente (não contínua) que ocorre durante períodos de sono. ^( 18 )^ **(** ***Modificado*** **)**
  **1.1.6.6 Incontinência urinária contínua:** queixa de perda de urina contínua. ^( 3 , 5 )^ **(** ***Modificado*** **)**
  **1.1.6.7 Incontinência urinária insensível:** queixa de incontinência urinária na qual o indivíduo tem consciência da perda de urina, mas não sabe como nem quando ela ocorre. ^( 5 )^ **(** ***Modificado*** **)**
  **1.1.6.8 Incontinência urinária postural:** queixa de incontinência urinária durante a mudança de postura ou de posição, como, por exemplo, de supino ou sentado para de pé. _NR 1.12_ **(** ***Novo*** **)**
  **1.1.6.9 Incontinência associada** à **incapacidade:** queixa de incontinência urinária quando há a incapacidade de se chegar ao banheiro no tempo, devido à incapacidade física (ortopédica e neurológica) e/ou mental. **(** ***Novo*** **)**
  **1.1.6.10 Incontinência por transbordamento:** queixa de incontinência urinária com presença de sintoma de bexiga muito (acima do normal) cheia (causa não identificada). **(** ***Novo*** **)**
  **1.1.6.11 Incontinência por excitação sexual:** queixa de perda involuntária de urina durante a excitação sexual, preliminares e/ou masturbação. **(** ***Novo*** **)**
  **1.1.6.12 Climatúria:** ^( 19 )^ queixa de perda involuntária de urina na hora do orgasmo.

### Síndrome de sintoma de armazenamento

**1.1.7 Síndrome da bexiga hiperativa (BH, urgência):** urgência miccional, geralmente acompanhada de aumento da frequência no decorrer do dia e/ou noctúria, com (BH úmida) ou sem (BH seca) incontinência urinária, na ausência de infecção do trato urinário (ITU) ou outra doença detectável. ^( 3 , 5 )^ **(** ***Modificado*** **)**

## SINTOMAS DE ESVAZIAMENTO

**1.2 Sintomas de esvaziamento:** sintomas do TUI durante a fase de esvaziamento (vivenciado durante a micção). **(** ***Novo*** **)**
  **1.2.1 Hesitação:** queixa de demora no início do esvaziamento (quando o indivíduo está pronto para urinar). **(** ***M*** ***odifi*** ***cado*** **)**
  **1.2.2 Parurese (“bexiga tímida”):** queixa da incapacidade de iniciar a micção em público (por exemplo, micção na presença de outras pessoas), apesar de não haver dificuldade quando sozinho. ^( 20 )^_NR 1.13_
  **1.2.3 Incapacidade episódica para esvaziar:** queixa de incapacidade ocasional para iniciar a micção apesar do relaxamento e/ou esforço intenso (por esforço abdominal, manobra de Valsalva ou pressão suprapúbica). **(** ***Novo*** **)**
  **1.2.4 Esforço miccional:** queixa da necessidade de se fazer um esforço intenso para iniciar, manter ou melhorar a micção ou fluxo urinário. ^( 3 , 5 )^ **(** ***Modificado*** **)**
  **1.2.5 Fluxo lento (urinário):** queixa de se notar um fluxo urinário mais lento do que micções anteriores ou se comparado com outros. ^( 3 , 5 )^
  **1.2.6 Intermitência:** queixa do fluxo urinário que para e começa em uma ou mais ocasiões durante um episódio de micção. ^( 3 , 5 )^
  **1.2.7** **Gotejamento terminal:** queixa de que, durante a parte final do esvaziamento, há diminuição perceptível do fluxo para gotas ou fluxo escorrendo. **(** ***Modificado*** **)**
  **1.2.8 Pulverização (divisão) do fluxo urinário:** queixa de que a passagem da urina é em *spray* ou dividida, ao invés de um único fluxo direcionado. ^( 3 , 5 )^ **(** ***Modificado*** **)**
  **1.2.9 Esvaziamento dependente da posição:** queixa de ter que adotar uma posição específica para conseguir esvaziar espontaneamente ou melhorar o esvaziamento da bexiga, como, por exemplo, precisar esvaziar na posição sentada. **(** ***Novo*** **)**
  **1.2.10 Disúria:** queixa de dor, ardência, outro desconforto ou dificuldade durante a micção. O desconforto pode ser intrínseco ao TUI (como, por exemplo, a bexiga ou uretra), externo, ou referido a outras estruturas adjacentes similarmente inervadas, como, por exemplo, ureter distal. _NR 1.14_ **(** ***Modificado*** **)**
  **1.2.11 Estrangúria:** queixa de micção lenta, difícil e espasmódica (às vezes, “gota a gota”) geralmente associada à dor. **(** ***Novo*** ) _NR 1.15_
  **1.2.12 Hematúria:** queixa de passagem de sangue misturado à urina. Isso pode ser no início, no fim ou durante todo o processo de esvaziamento da bexiga. **(** ***Novo*** **)**
  **1.2.13 Pneumatúria:** ^( 1 )^ queixa da passagem de gás (ou ar) pela uretra durante ou depois da micção. **(** ***Novo*** **)**
  **1.2.14 Fecalúria:** ^( 1 )^ queixa da passagem de fezes (pela uretra) na urina. **(** ***Novo*** **)**
  **1.2.15 Quilúria (albidúria):** ^( 1 )^ queixa da passagem de líquido claro ou branco, cor leite (quiloso) na urina. ^( 1 )^ **(** ***Novo*** **)**
  **1.2** **.16 Rete** **nção urinária:** queixa da incapacidade de esvaziar completamente a bexiga. ^( 1 )^ **(Novo)**
    **1.2.16.1 Retenção urinária aguda:** queixa de um rápido começo, geralmente uma sensação suprapúbica dolorosa (em razão de uma bexiga cheia) por causa da incapacidade de esvaziar (não episódico), apesar de um esforço intenso e persistente. _NR 1.16_ **(** ***Novo*** **)**
    **1.2.16.2 Retenção urinária crônica:** queixa de uma incapacidade crônica ou repetida para esvaziar a bexiga, apesar de conseguir urinar um pouco. Isso pode ser o resultado da eliminação frequente de pequenas quantidades de urina ou incontinência urinária e uma bexiga distendida. _NR 1.17_ **(** ***Novo*** **)**

## SINTOMAS PÓS-MICCIONAIS

1.3 Sintomas pós-miccionais**: sintomas do TUI sentidos depois que o esvaziamento cessou. **(** ***Novo*** **)**
  1.3.1 Sensação de esvaziamento incompleto (bexiga**): queixa de sentir que a bexiga não está vazia após a micção ter cessado. ^( 3 , 5 )^ **(** ***Modificado*** **)**
  1.3.2 Necessidade imediata de novo esvaziamento (“bis” ou esvaziamento “duplo”)**: Queixa de que um novo esvaziamento é necessário logo após a micção (término do fluxo). ^( 3 , 5 )^ **(** ***Modificado*** **)**
  1.3.3 Incontinência pós-miccional**: queixa de uma nova micção **involuntária** (incontinência) ou gotejamento logo após o término da micção. ^( 3 , 5 )^_NR 1.18_ **(** ***Novo*** **)**
  **1.3.4 Urgência pós-miccional:** queixa de urgência persistente após a micção. **(** ***Novo*** **)**

### Síndrome dos sintomas de esvaziamento (proposta para futuras pesquisas) Síndrome da bexiga hipoativa: NR 1.19

**1.4** Dor no trato urinário inferior e/ou outra dor pélvica
  **1.4.1 Dor:** sensação desagradável e inconstante. ^( 1 )^ Pode ser descrita pelo paciente como pressão ou desconforto. A dor deve ser caracterizada por local, tipo, frequência, duração, precipitação e fatores de alívio. _NR 1.20__,__NR 1.21_ **(** ***Modificado*** **)**
  **1.4.2 Dor na bexiga:** queixa de dor, pressão, desconforto suprapúbicos ou retropúbicos, relacionados à bexiga e, geralmente, associados ao enchimento dela. A dor pode persistir ou ser aliviada após a micção. ^( 3 , 5 )^ **(** ***Modificado*** **)**
  **1.4.3 Dor uretral:** queixa de dor, pressão ou desconforto sentido na uretra ^( 3 , 5 )^ antes, durante e/ou depois da micção, e o homem indica a uretra como o local. **(*Modificado* )**
  **1.4.4 Dor escrota** **l:** queixa de dor, pressão ou desconforto sentido no escroto ou ao redor dele. ^( 3 )^ Ela pode ser localizada nos testículos, epidídimo, estrutura dos cordões ou pele do escroto.
  **1.4.5 Dor perineal:** queixa de dor, pressão ou desconforto sentida na superfície ou no interior do tecido entre o escroto e o ânus. ^( 3 )^
  **1.4.6 Dor pélvica:** queixa de dor, pressão ou desconforto relacionado à pelve, mas que não está claramente relacionada à bexiga, à uretra, ao escroto ou ao períneo. ^( 3 )^
  **1.4.7 Dor na ejaculação:** queixa de dor, pressão ou desconforto sentida no períneo, região suprapúbica e/ou pênis durante a ejaculação, mas ela pode continuar por mais algum tempo após o ato de ejacular. **(** ***Novo*** **)**_NR 1.22_
  **1.4.8 Sintomas de dor anorretal:** queixa de dor, pressão ou desconforto particularmente durante a defecação ou força para defecar, mas ela pode ocorrer a qualquer momento. **(** ***Novo*** **)**
    **1.4.8.1 Dor durante o esforço/defecação:** dor durante a defecação ou esforço para defecar.
    **1.4.8.2 Dor anorretal inflamatória:** dor caracterizada por ardência ou ferroada (inflamação, radiação e sepse). **(** ***Novo*** **)**
    **1.4.8.3 Dor anorretal não inflamatória:** dor anorretal mais fraca (proctalgia tímida, síndrome do levantador do ânus e neuralgia do pudendo). **(** ***Novo*** **)**
    **1.4.9 Dor coccígea (coccidínia):** queixa de dor, pressão ou desconforto na região coccígea. **(** ***Novo*** **)**
    **1.4.10 Dor pudenda: (neuralgia):** queixa de dor, pressão ou desconforto em uma ou mais áreas inervadas pelo nervo pudendo (pode ser causada pela inflamação ou pelo aprisionamento do nervo pudendo envolvendo seu dermátomo). **(** ***Modificado*** **)**
    **1.4.11 Síndrome da dor pélvica crônica:** ver padronização da ICS para a terminologia da síndrome da dor pélvica crônica. ^( 23 )^_NR 1.21_
**1.5 Infecção do trato urinário (ITU):**
  **1.5.1** **Sintomas de infecção aguda do trato urinário:** sintomas como aumento da sensibilidade da bexiga, urgência e frequência, disúria/estrangúria, dor no TUI com ou sem incontinência urinária de urgência podem sugerir. A confirmação de ITU requer evidência de micro-organismos e piúria. _NR 1.23, NR 1.24_ **(** ***Modificado*** **)**
  **1.5.2 Infecções recorrentes do trato urinário (ITUr):** uma história de pelo menos dois diagnósticos clínicos de infecções sintomáticas do trato urinário nos últimos 12 meses. _NR 1.25_ A última ITU deve ter sido resolvida antes que uma nova seja diagnosticada. **(** ***Modificado*** **)**
  **1.5.3 S** **ecreção uretral:** de muco, pus ou sangue pelo meato uretral. **(** ***Novo*** **)**
**1.6 Sintomas de disfunção sexual:** sensação e/ou função anormal relatada pelo homem durante a atividade sexual. **(** ***Novo*** **)**
  **1.6.1 Libido alterada:** mudança de interesse na atividade sexual. **(** ***Novo*** **)**
  **1.6.1.1 Diminuição da libido:** queixa da diminuição do interesse pela atividade sexual em comparação com experiências anteriores. **(** ***Novo*** **)**
  **1.6.1.2 Aumento da libido:** queixa de aumento do interesse pela atividade sexual em comparação com experiências anteriores. **(** ***Novo*** **)**
  **1.6.2 Disfunção erétil:** ^( 25 )^ queixa de incapacidade para atingir e sustentar a ereção firme o suficiente para um desempenho sexual satisfatório. **(** ***Novo*** **)**
  **1.6.3 Disfunção ejaculatória:** queixa de alteração da emissão de líquidos seminais durante a ejaculação. **(** ***Novo*** **)**
    **1.6.3.1 Anejaculação:** queixa de falta de emissão de líquido seminal. Pode ser associado à ausência de sensação de orgasmo ou anorgasmia. **(** ***Novo*** **)**
    **1.6.3.2 Ejaculação retardada:** queixa do aumento do tempo que leva para a ejaculação ocorrer. **(** ***Novo*** **)**
    **1.6.3.3 Ejaculação precoce:** queixa de padrão recorrente ou persistente de ejaculação muito rápida durante a atividade sexual em parceria, ^( 1 )^ ou seja, antes que o indivíduo deseje ejacular. _NR 1.26_ **(** ***Novo*** **)**
    1.6.3.4 Redução (baixa) do volume de sêmen**: queixa de uma quantidade de líquido seminal menor que o normal ou visto anteriormente. _NR 1.27_ **(** ***Novo*** **)**
    **1.6.3.5 Aumento do volume (alto) de sêmen:** queixa de uma quantidade de líquido seminal maior que o normal ou visto anteriormente. **(** ***Novo*** **)**_NR 1.27_
  **1.6.4 Hematospermia:** queixa do aparecimento visível de sangue no líquido seminal. A cor do líquido seminal pode ser vermelha ou marrom. **(** ***Novo*** **)**
  **1.6.5 Dor peniana durante a relação sexual (dispareunia masculina):** queixa de qualquer desconforto peniano que ocorre durante a relação. Pode ser causada por doença peniana, anatomia vaginal (por exemplo: estreitamento vaginal, cicatrizes ou tela exposta) e/ou pode ser relacionada a várias posições durante a relação. _NR 1.28_ **(** ***Novo*** **)**
  **1.6.6 Coito obstruído:** queixa de que a relação não é possível devido a uma obstrução que foi percebida. Isso pode ser um problema da parceira, mas também pode ocorrer em casos de curvatura peniana (doença de Peyronie) ou carcinoma peniano. **(** ***Novo*** **)**
**1.7 Sintomas de disfunção anorretal:** ^( 5 , 10 , 27 )^_NR 1.29_
  **1.7.1 Incontinência anorretal (sintomas):** queixa de perda involuntária de flatos ou fezes. ^( 5 , 10 )^ Pode ainda ser dividida em:
    **1.7.1.1 Incontinência para flatos:** queixa de perda involuntária de flatos (gases). ^( 5 , 10 )^
    **1.7.1.2 Incontinência fecal:** queixa de perda involuntária de fezes. ^( 5 , 10 )^
- quando as fezes são sólidas e/ou
- quando as fezes são líquidas
  **1.7.1.3 Urgência fecal (retal):** queixa de um repentino e imperativo desejo de defecar que é difícil de adiar. ^( 5 , 10 )^
  **1.7.1.4 Incontinência fecal (flatos) de urgência:** queixa de perda involuntária de fezes (flatos) associada à urgência fecal. ^( 5 , 10 , 27 )^
    **1.7.1.5 Incontinência fecal passiva (insensível):** queixa de sujidade involuntária de fezes líquidas ou sólidas sem sensação ou aviso. **(** ***Novo*** **)**
    **1.7.1.6 Incontinência fecal de transbordamento:** queixa de perda involuntária de fezes devido a um reto excessivamente cheio ou fecaloma. **(** ***Novo*** **)**
    **1.7.1.7 Incontinência fecal durante o coito:** queixa de perda involuntária de fezes durante a relação sexual. ^( 5 , 10 )^
      **1.7.1.8 Incontinência fecal por esforço:** queixa de perda involuntária de fezes durante esforços físicos, incluindo atividades esportivas ou espirros ou tosses. **(** ***Novo*** **)**
        **1.7.2 Sintomas sensoriais anorretais**
          **1.7.2.1** **Diminuição sensação retal (hipossen** **sibilidade retal):** queixa de falta ou diminuição da sensação de enchimento do reto **. (** ***Modificado*** **)**
          **1.7.2.2 Aumento da sensação retal (hipersensibilidade retal):** queixa de desejo de defecar (durante o enchimento do reto), que ocorre antecipadamente ou com mais persistência do que vivenciado anteriormente. **(** ***Novo*** **)**
          **NB**: para itens 1.7.2.1 e 1.7.2.2, pode ser (i) primeira sensação, (ii) sensação de urgência e (iii) volume máximo tolerado.
          **1.7.2.3 Tenesmo:** queixa de desejo urgente de evacuar o intestino, acompanhado de esforço involuntário e passagem de pouco material fecal. ^( 1 )^
        **1.7.3 Sintomas defecatórios ou pós-defecatórios:** sintomas sentidos durante ou após o ato de defecar. **(** ***Novo*** **)**
          **1.7.3.1 Constipação:** queixa de que o hábito intestinal é infrequente e/ou incompleto e/ou há a necessidade de esforço ou ajuda manual para defecar (critérios Roma IV). ^( 28 )^_NR 1.30_
          **1.7.3.1.1 Trânsito lento:** hábito intestinal infrequente, devido ao retardo do conteúdo do intestino em alcançar o reto.
          **1.7.3.1.2 Defecação obstruída:** queixa da dificuldade em evacuar devido à obstrução mecânica.
          **1.7.3.2 Sensação de evacuação incompleta do intestino:** queixa de sentir que o reto não está vazio após a defecação. Pode ser acompanhado de um novo desejo de evacuar. ^( 5 , 10 )^
          **1.7.3.3 Esforço para defecar:** queixa de que é necessário fazer um esforço intenso (por esforço abdominal ou Valsalva) ou usar massagem abdominal para iniciar, manter ou melhorar a defecação. ^( 5 , 10 )^
          **1.7.3.4 Assistência defecatória manual:**
          **1.7.3.4.1 Interna:** **digitação anorretal:** queixa de que é necessário usar os dedos no reto para ajudar manualmente a evacuação do conteúdo das fezes por escavação, alongamento e/ou estimulação. ^( 5 )^ **(** ***Novo*** **)**
          **1.7.3.4.2 Externa:** **pressão perineal ou separação das nádegas:** queixa da necessidade de pressionar o períneo ou separar as nádegas para ajudar na defecação. **(** ***Novo*** **)**
          **1.7.3.5 Sujidade pós-defecatória:** queixa de sujidade que ocorre depois da defecação. **(** ***Novo*** **)**
          **1.7.3.6 Sangramento/muco retal**: queixa de perda de sangue ou muco pelo reto.
        **1.7.4 Prolapso anorretal:** queixa de protusão (protuberância) externa do ânus ou reto (diferenciação no exame subsequente entre o prolapso da mucosa retal e o da parede retal e a espessura total, que inclui camadas musculares e serosa). **(** ***Modificado*** **)**
**1.8 Outra história relevante** Medicamentos atuais, cirurgias urológicas anteriores, radioterapia e cateterismo devem ser observados e levados em conta.

## Notas de rodapé da seção 1

1.1: Milsom et al. ^( 15 )^ reportaram primeiramente que a frequência causada por uma BH foi arbitrariamente definida como mais que oito micções no período de 24 horas, considerando que a frequência de esvaziamento normal em indivíduos saudáveis é tipicamente inferior a seis micções a cada 24 horas. Foi maior do que previamente relatado para mulheres saudáveis usando uma tabela de frequência/volume (mediana de 5,5 micções a cada 24 horas). ^( 15 - 17 )^

1.2: Polaciúria: queixa de frequência anormal de micção (extraordinária; ^( 1 )^ definição raramente usada).

1.3: O esvaziamento à noite é comum quando o sono é interrompido por outras razões (por exemplo: insônia). Isso não constitui noctúria.

1.4: Poliúria é mais completamente definida na seção “sinais”.

1.5: “Urgência” substitui “impulso” como terminologia “aceita” para fenômenos anormais ao normal.

1.6: O uso de “súbito”, definido como “sem aviso abrupto”, usado nas definições anteriores, ^( 2 , 3 , 5 )^ tem sido assunto de muito debate. Sua inclusão foi continuada.

1.7: Esse sintoma geralmente ocorre se houver alguma forma de doença neurológica.

1.8: “Continência” é definida como ausência de vazamento involuntário do conteúdo do intestino ou bexiga. (por exemplo, controle voluntário normal da função do intestino e da bexiga).

1.9: Em cada circunstância específica, incontinência urinária deveria ser descrita especificando fatores relevantes, como tipo, severidade, fatores precipitantes, impacto social, efeito sobre a higiene, qualidade de vida, medidas adotadas para conter o vazamento e se o indivíduo procura ou deseja ajuda por causa da incontinência urinária ou não.

1.10: Essa mudança é para ajustar a ambiguidade em algumas línguas entre ansiedade e estresse. Esse sintoma deveria ocorrer mais comumente em homens que se submeteram à prostatectomia (radical). Homens que passaram por prostatectomia radical podem vir a ter incontinência relacionada à atividade e/ou durante o sexo. ^( 19 )^

1.11: Pequenas quantidades de urina podem vazar sem aviso.

1.12: Homens com incontinência pós-prostatectomia realmente relatam isso. Isso também acontece depois que homens se submetem à colocação do esfíncter artificial. Quando eles levantam, têm vazamento. Pode ser devido ao esforço e sem urgência ou outros sintomas associados a levantar-se ou à posição ereta.

1.13: O termo “parurese” não é de uso comum, embora o sintoma seja bem conhecido. ^( 20 )^ Parurese é definida como um medo de ser capaz de urinar em situações em que outras pessoas estão presentes. Diagnóstico e manual estatístico de desordens mentais. Arlington, VA: Associação Americana de psiquiatria; 2013.

1.14: Disúria é um tipo de dor uretral, mas pode ser uretral pela origem ou se referindo a ela a partir de um processo patológico na bexiga, no ureter distal ou na próstata.

1.15: O sintoma de “estrangúria” é pouco entendido, sobrepondo-se, às vezes, à dor uretral, à disúria e à dor pélvica.

1.16: Retenção urinária aguda a bexiga é distendida, palpável e possivelmente dolorida. Um resíduo significativamente aumentado está presente.

1.17: Retenção urinária crônica não neurogênica em homens (consenso da AUA, ^( 21 )^ apoiado pelos autores atuais) pode ser definida como elevado resíduo pós-miccional (RPM) maior que 300mL que persistiu por, no mínimo, 6 meses e é documentado em duas ou mais situações diferentes. A evidência não é forte. Retenção urinária crônica pode ser causada por diferentes patologias que promovem hipoatividade do detrusor (HAD) e/ou resultam de obstrução infravesical (OIV) crônica. ^( 21 )^

1.18: Pode ocorrer após o ajuste de roupas por causa de algum “acúmulo” de urina na uretra se a roupa íntima ou vestuário causou alguma obstrução durante a micção ou estenose ou divertículo da uretra.

1.19: Queixa de fluxo urinário lento, hesitação e força para esvaziar, com ou sem sensação de esvaziamento incompleto da bexiga e gotejamento, às vezes com sintomas de armazenamento: o agrupamento dos sintomas proposto é sugestivo de HAD. ^( 22 )^ O diagnóstico de hipoatividade real do detrusor depende dos achados urodinâmicos, como discutido na seção 5, em diagnósticos.

1.20: É frequentemente difícil localizar precisamente a dor, então descrições, como a localização da dor, podem ser imprecisas. Por exemplo, o termo “dor na bexiga” não necessariamente indica que a bexiga é a causa. Uma dor que se pensa surgir na bexiga ou ser sentida na uretra, no escroto ou no períneo pode ser referente ao ureter distal, à base da bexiga ou outros órgãos pélvicos.

1.21: As definições de dor pélvica e, especialmente, de dor pélvica crônica têm sido debatidas em várias sociedades, com vistas à simplificação e à reestruturação da classificação. A ICS publicou recentemente um relatório de síndromes de dor pélvica crônica (SDPC). ^( 23 )^

1.22: Ejaculação dolorida (anteriormente chamada de “odinorgasmia”) é uma síndrome pobremente caracterizada. Pode ser associada com uretrite, HPB, prostatite crônica ou aguda, SDPC, vesiculite seminal, cálculos vesiculares seminais ou obstrução do duto ejaculatório. Frequentemente nenhum fator etiológico óbvio pode ser encontrado.

1.23: Critérios comumente sugeridos para (i) bacteriúria são >100.000CFU/mL em espécimes urinados ou >1.000CFU/mL em espécimes caracterizados; (ii) para piúria, são >10WBC/mm ^3^ na urina não centrifugada. A presença de nitritos na urina é favorável a uma ITU envolvendo um organismo comum ( *Escherichia coli* e *Klebsiella* ).

1.24: Esses pacientes sintomáticos com menos contagem de colônias ainda podem abrigar organismos detectáveis por análises de mRNA, que não estão amplamente disponíveis no momento. ^( 24 )^ Teste para microbioma urinário está sendo explorado, mas não está amplamente disponível.

1.25: As infecções do trato urinário (ITUs) não foram consistentemente definidas. Elas são menos comuns em homens do que em mulheres, mas, talvez, mais significantes. Há dificuldade em equilibrar a definição clínica prática e a científica. Registros de testes de diagnósticos são, muitas vezes, inacessíveis a médio e longo prazo. Tendendo para a primeira categoria, uma definição poderia ser a presença de, pelo menos, duas das ITUs sintomáticas e diagnosticadas clinicamente em 12 meses. “Recorrer” significa rigorosamente “ocorrer de novo” ou “ser repetida”.

1.26: Esse sintoma deve estar presente por, no mínimo, 6 meses e ser sentido em quase todas ou todas (aproximadamente 75% a 100%) ocasiões de atividade sexual. Clinicamente, isso causa significante angústia no indivíduo. É chamado de ejaculação precoce, ejaculação rápida, clímax rápido ou clímax prematuro. Não há um ponto de marcação específico para definir “precoce”, mas um consenso de especialistas da *International Society for Sexual Medicine* endossou a definição de cerca de 1 minuto depois da penetração. A Classificação Internacional de Doenças e Problemas Relacionados à Saúde (CID-10) usa um ponto de marcação de 15 segundos do começo da relação sexual.

1.27: O volume médio de sêmen é 3,9mL (quinto percentil: 1,5mL; 95º percentil: 6,8mL). Baixo volume de sêmen é abaixo de 1,5mL; alto volume de sêmen é acima de 6,8mL. ^( 25 , 26 )^

1.28: Dispareunia (“hispareunia”), o sintoma mais aplicável ao desconforto do homem na relação sexual depende de muitos fatores, incluindo o relaxamento do introito da mulher e/ou fatores anatômicos.

1.29: Sintomas da disfunção defecatória não são incomuns em homens, particularmente naqueles que se submeteram a esfincterotomias por fissuras anais.

1.30: Os critérios de Roma IV para **item 1.8.3.1** Constipação: ^( 28 )^ queixa de que o hábito intestinal é (i) infrequente (<3 por semana); (ii) necessidade de esforçar; (iii) fezes grumosas, duras e entumecidas; (iv) sensação de evacuação incompleta; (v) sensação de obstrução anorretal ou dor abdominal obstrutiva e (vi) necessidade de ajuda manual em mais do que um quarto de toda a defecação.

## SEÇÃO 2 – SINAIS

**Sinal:** Qualquer anormalidade indicativa de doença ou problema de saúde, detectável no exame do paciente; uma indicação objetiva de doença ou problema de saúde. ^( 1 )^ **( *Modificado* )**

**Princípios gerais de exame para homens com sintomas de disfunção do TUI/AP:** ^( 29 )^

O exame físico abrangente é feito para buscar possíveis influências nos sintomas. _NR 2.1_ Deve incluir o exame abdominal, focando a região suprapúbica, para detectar bexiga distendida ou outro tumor abdominal, e o exame digital retal (próstata), bem como da genitália externa, do períneo e dos membros inferiores. Os orifícios de hérnia também devem ser avaliados. Lesões penianas, incluindo estenose meatal, fimose e câncer de pênis, devem ser excluídas. ^( 29 - 31 )^

Se houver suspeita de diagnóstico neurológico, precisará ser realizado exame neurológico com foco na avaliação da sensibilidade perianal pontiaguda e romba. Além disso, o tônus muscular anal pode ser avaliado com o dedo no reto e pedindo ao paciente para apertar. **( *Novo* )**

**2.1 Observações gerais (visuais)**
  **2.1.1 Mobilidade:** força muscular generalizada e capacidade para deambular de forma independente ou com assistência. **(** ***Novo*** **)**
  **2.1.2 Pele:** icterícia ou palidez ou irritação da pele, devido à perda urinária. **(** ***Novo*** **)**
  **2.1.3 Estado nutricional:** caquexia (possível malignidade subjacente); obesidade (possível anormalidade endócrina, ^( 29 )^ incluindo síndrome metabólica). **(** ***Novo*** **)**
  **2.1.4 Edema de órgãos genitais e extremidades inferiores:** possível descompensação cardíaca, insuficiência renal, síndrome nefrótica ou obstrução linfática retroperitonal e/ou pélvica. ^( 29 - 31 )^ **(** ***Novo*** **)**
  **2.2 Exame abdominal:** ^( 3 , 5 )^ entre numerosos sinais abdominais possíveis, temos:
  **2.2.1 Plenitude/retenção vesical:** a bexiga pode ser sentida por palpação abdominal ou detectada por percussão suprapúbica. _NR 2.2_ ***(Modificado*** **)**
  **2.2.2 Outros tumores abominais:** ou distensão (por exemplo, ascites). **(** ***Novo*** **)**
  **2.2.3 Cicatrizes:** indicando cirurgia relevante anterior, traumas, ou evidência de radioterapia prévia. **(** ***Novo*** **)**
  **2.2.4 Área renal:** exame para sensibilidade, tumor. **(** ***Novo*** **)**
  **2.3 Trato urinário inferior/exame genital/sinais**
  **2.3.1 Pele genital:**
    **2.3.1.1** Escoriação, vermelhidão, irritação secundária à incontinência urinária e marcas de absorventes ou fraldas. **(** ***Novo*** **)**
    **2.3.1.2** Infecções micóticas (balanopostite, intertrigo ou escrotal): pele úmida, vermelha e pruriginosa, geralmente em homens com incontinência urinária ou fecal, imunossupressão ou *diabetes mellitus* mal controlada. ^( 32 )^ **(** ***Novo*** **)**
    **2.3.1.3** Pigmentação da pele: balanite xerótica obliterante (sinônimo líquen escleroso) e vitiligo podem causar despigmentação (pele peniana, escroto e glande). **(** ***Novo*** **)**
    **2.3.1.4** Manifestações cutâneas de doenças sexualmente transmissíveis: vesículas e úlceras. **(** ***Novo*** **)**
    **2.3.2 Exame do pênis:**
    **2.3.2.1** Anormalidades do prepúcio:
    **2.3.2.1.1** Tumor ou infecção (balanopostite), isto é, inflamação da glande e da pele do prepúcio (tumor maligno do pênis que acomete o prepúcio ou infecção). ^( 1 )^ **(** ***Novo*** **)**
    **2.3.2.1.2 Fimose:** ^( 33 )^ incapacidade parcial ou total de retrair o prepúcio, devido à adesão entre a glande e o prepúcio ou anel prepucial. _NR 2.3_ **(** ***Novo*** **)**
    **2.3.2.1.3 Parafimose:** ^( 33 )^ estrangulamento do prepúcio por trás da glande. _NR 2.3_ **(** ***Novo*** **)**
    **2.3.2.2 Posição do meato uretral:** ^( 31 )^
    **2.3.2.2.1 Hipospádias:** refere-se ao meato uretral localizado na **face ventral** do pênis, congênito ou adquirido, próximo à sua posição normal na ponta da glande. O meato uretral externo pode estar na glande do pênis (hipospádia glandar), no sulco (hipospádia coronal), na haste (hipospádia peniana), no escroto (hipospádia escrotal) ou no períneo (hipospádia perineal). **(** ***Novo*** **)**
    **2.3.2.2.2 Epispádias:** refere-se ao meato uretral situado na **superfície dorsal** do pênis, seja congênito ou adquirido, proximal à sua posição normal na ponta da glande. **(** ***Novo*** **)**
    **2.3.2.2.3 Lesões neoplásicas ou inflamatórias** no interior da fossa navicular. ^( 34 )^ **(** ***Novo*** **)**
    **2.3.3.3.4 Correção pós-hipospádia/epispádia, incluindo fibrose uretral pós-uretroplastia:** palpada próxima ao meato ou no pênis. **(** ***Novo*** **)**
    **2.3.2.2.5 Fístula pós-operatória:** a urina é visível nas linhas de incisão ou perto delas. **(** ***Novo*** **)**
  **2.3.2. Exame uretral**
    **2.3.2.3.1 Palpação:** ao longo do aspecto ventral do pênis e inferiormente, no períneo, para detectar fibrose, caroços ou sensibilidade ao longo da haste. **(** ***Novo*** **)**
    **2.3.2.3.2 Sensibilidade:** sugestiva de inflamação uretral ou periuretral, muitas vezes secundária à estenose de uretra ^( 34 )^ ou à doença sexualmente transmissível. **(** ***Novo*** **)**
    **2.3.2.3.3 Estenose meatal:** alterações com estreitamento da uretra distal; pós-infecção, pós-operatória. **(** ***Novo*** **)**
    **2.3.2.4 Exame da glande e da haste**
    **2.3.2.4.1 Placa peniana:** palpação de nódulo ou placa na túnica, geralmente no face dorsal (talvez relacionada à doença de Peyronie). **(** ***Novo*** **)**
    **2.3.2.4.2 Líquen escleroso:** prepúcio apertado, rachaduras e sangramento.
    **2.3.2.5 Exame geral:** vermelhidão, úlceras e verrugas. **(** ***Novo*** **)**
    **2.3.3 Exame escrotal (** ***Novo*** **)**
    **2.3.3.1 Normal:** o escroto é um saco solto contendo os testículos e estruturas do cordão espermático. O epidídimo é palpável, localizado na superfície posterior do testículo, como uma crista, embora ocasionalmente esteja situado na superfície anterior. _NR 2.4_ **(** ***Novo*** **)**
    **2.3.3.2 Inflamação:** o epidídimo pode estar inchado e sensível e, se for grave, o processo inflamatório pode envolver todo o conteúdo escrotal (ou seja, testículos e epidídimos – orquiepididimite) e a pele escrotal também. _NR 2.5_ **(** ***Novo*** **)**
    **2.3.3.3 Dilatações císticas do epidídimo:** (cistos epididimários ou espermatocele) e hidroceles (coleções líquidas entre a túnica albugínea visceral e a camada parietal do peritônio testicular): são geralmente benignas. O exame dessas estruturas seria geralmente não sensível e sem dor (em oposição ao **item** **2.3.3.2** ). _NR 2.6_ **(** ***Novo*** **)**
    **2.3.3.4 Abaulamento inguinal:** exame e diferenciação de hérnia de hidrocele ou cisto de cordão espermático ou linfonodos inguinais. **(** ***Novo*** **)** (O uso de transiluminação pode ajudar, embora a ultrassonografia seja geralmente diagnóstica). _NR 2.7_
  **2.3.4 Exame perineal:** geralmente é realizado com o paciente na posição supina lateral ou de litotomia. **(** ***Novo*** **)**
    **2.3.4.1 Dermatite perianal:** infecção da pele no períneo ao redor do ânus, geralmente associada à incontinência fecal ou à diarreia. **(Novo)**
    **2.3.4.2 Fissuras:** ruptura ou corte na pele do períneo, esfíncter anal ou reto distal, geralmente associada à dor anal. **(** ***Novo*** **)**
  **2.3.5 Exame re** **tal e da próstata:** o exame digital retal é recomendado ^( 35 - 37 )^ como parte do exame físico. Geralmente, é feito com o paciente em pé e inclinado sobre a mesa de exame, ou na posição lateral esquerda, com joelhos fletidos, ou de litotomia. Na maioria das vezes, é realizado sem dor. **(** ***Novo*** **)**
    **2.3.5.1 Exame anal:** pode detectar os seguintes achados no esfíncter anal ou no reto distal: **(** ***Novo*** **)**
    **2.3.5.1.1 Doenças benignas:** hemorroidas, fissura, lesão do esfíncter anal, desconforto no músculo elevador ou dor. **(** ***Novo*** **)**
    **2.3.5.1.2 Possíveis doenças malignas:** carcinoma anal, distal do reto e próstata. **(** ***Novo*** **)**
    **2.3.5.1.3 Tônus anal:** o tônus do esfíncter anal aumentado ou diminuído pode sugerir alterações semelhantes no esfíncter urinário e indicar doença neurológica. **(** ***Novo*** **)**
    **2.3.5.1.4 Estenose anal:** estreitamento ou estenose circunscrita do canal anal. **(** ***Novo*** **)**
    **2.3.5.2** **Características da glândula prostática:** tamanho, simetria, consistência, nódulos e sua relação com a parede pélvica e o reto podem ser avaliados. ^( 35 - 37 )^ A glândula tem aproximadamente o tamanho de uma noz e consistência semelhante à da eminência tenar do polegar contraída. _NR 2.8_ **(** ***Novo*** **)**
    **2.3.5.3 Nodularidade e/ou firmeza:** pode indicar possível anormalidade que requeira investigação adicional. ^( 37 )^ **(** ***Novo*** **)**
    **2.3.5.4 Sensibilidade da próstata:** a palpação da próstata, como parte do toque retal, é geralmente indolor. A dor com palpação prostática é variável, embora esteja presente, pode ser útil na diferenciação das síndromes da próstata/dor pélvica. ^( 34 )^ **(** ***Novo*** **)**
    **2.3.5.5 Exame retal (circunferencial):** pode levar à detecção de doenças não urológicas, como carcinoma retal, fístula e impactação fecal. _NR 2.9_ **(** ***Novo*** **)**
**2.4 Exame neurológico focalizado:** ^( 3 , 5 )^
  **2.4.1 Estado neurológico geral:** anormalidades da fala, da marcha e da destreza dos membros superiores e inferiores devem ser notadas, pois podem indicar causa neurológica para os sintomas urológicos. Neuropatia pode interferir também nas opções de tratamento. ^( 38 )^_NR 2.10_ **(** ***Modificado*** **)**
  **2.4.2 Nível** **da anormalidade neurológica:** pode ocasionalmente ser localizada de acordo com o padrão de *deficit* sensitivo ou motor observado durante o exame físico, utilizando um mapa de dermátomo. ^( 30 )^ **(** ***Novo*** **)**
  **2.4.3** ***Deficit*** **sensoriais do pênis, escroto ou perianal:** podem indicar danos ou lesões nas raízes ou nos nervos sacrais. O teste de reflexos na área genital também pode ser realizado. O mais importante deles é o reflexo bulboesponjoso. ^( 39 )^ **(** ***Novo*** **)**
  **2.4.4 Reflexo bulboesponjoso:** ^( 39 )^ contração reflexa do músculo estriado do AP (esfíncter anal) e do músculo bulbo esponjoso, que ocorre em resposta a vários estímulos no períneo ou genitália. _NR 2.11_ **(** ***Novo*** **)**
  **2.4.5. Reflexo cremastérico:** contração do músculo cremaster ipsilateral, tracionando o testículo para cima, quando a parte interna e superior da coxa é tocada longitudinalmente. **(** ***Novo*** **)**
**2.5 Sinais de incontinência urinária:** ^( 3 , 5 , 40 , 41 )^ todos os exames para avaliação da incontinência urinária são melhores realizados com a bexiga do indivíduo confortavelmente cheia.
  **2.5.1** **Incontinência urinária:** ^( 3 , 5 )^ observação de perdas involuntárias de urina ao exame.
  **2.5.2 Incontinência urinária de esforço (perda clínica por esfor** ç **o):** ^( 3 , 5 )^ observação de perda involuntária de urina pelo meato uretral, ao mesmo tempo do esforço ou esforço físico, ou com espirro ou tosse.
  **2.5.3 Incontinência urinária de urgência:** ^( 3 , 5 )^ observação de perda de urina involuntária pelo meato uretral, associada ao desejo súbito e compulsivo de esvaziar a bexiga, relatado pelo indivíduo. **(** ***Modificado*** **)**
  **2.5.4 Incontinência extrauretral:** ^( 3 , 5 )^ observação de vazamento de urina através de outros canais, que não o meato uretral, como, por exemplo, fístula. _NR 2.12_
**2.6 Função da musculatura do AP (MAP).** ^( 3 , 5 , 42 , 43 )^ Os seguintes sinais da função da MAP podem ser avaliados por meio do exame do períneo (exame visual ou tátil) ou do reto (palpação digital). O exame digital retal pode ser menos útil nas disfunções urinárias masculinas, em que o esfíncter uretral, inacessível ao toque retal, tem papel mais importante. ^( 44 )^ **(** ***Novo*** **)**
  **2.6.1 Exame perineal:** ^( 3 , 5 , 42 , 43 )^ quando o paciente é solicitado a tossir ou abaixar, o períneo deve mostrar apenas um movimento descendente limitado. Movimento ventral pode ocorrer por causa das ações de proteção dos músculos do AP. **(** ***Modificado*** **)**
    **2.6.1.1 Elevação perin** **eal:** ^( 43 , 44 )^ é o movimento interior (ventro-cefálico) do períneo e do ânus. _NR 2.13_ Procure por elevação testicular e retração peniana. Estas precisam ser verificadas contra o movimento do escroto e do pênis inteiro. O movimento correto ocorre apenas com a MAP: o eixo do pênis entra e os testículos elevam-se, em direção cefálica. Esses movimentos podem ser melhor visualizados em pé do que na posição supina. ^( 45 - 47 )^ **(** ***Novo*** **)**
    **2.6.1.2 Descida perineal:** ^( 43 )^ este é o movimento externo (dorso-caudal) do períneo e do ânus.
  **2.6.2 Exames** ^( 43 )^
    **2.6.2.1** **Estado da MAP em repouso:** aspectos a avaliar. _NR 2.13_
    **2.6.2.1.1 Mialgia:** provocada pela palpação. A dor/tensão muscular do elevador pode ser desencadeada pela palpação desses músculos, por meio de exame retal. ^( 43 )^_NR 2.14_ **(** ***Novo*** **)**
    **- Ponto sensível:** sensibilidade à palpação em um local específico do tecido mole. **(** ***Novo*** **)**
      **2.6.2.1.2 Tônus:** estado do músculo, geralmente definido por sua tensão de repouso, clinicamente determinado pela resistência ao movimento passivo. O tônus muscular tem dois componentes: o contrátil e o viscoelástico. O tônus muscular pode ser alterado na presença ou na ausência de dor. **(** ***Modificado*** **)**
      **2.6.2.1.3 Aumento do tônus da MAP (hipertonicidade não neurogênica):** aumento do tônus em paciente sem diagnóstico neurológico intercorrente. **(** ***Modificado*** **)**
      **2.6.2.1.4 Diminuição da MAP (hipotonicidade não neurogênica):** diminuição do tônus em paciente sem diagnóstico neurológico intercorrente. **(** ***Modificado*** **)**
      **2.6.2.1.5 Simetria:** quando examinar o paciente em decúbito lateral esquerdo, haverá um efeito de gravidade, e o lado correspondente terá uma sensação diferente do lado superior. Os dois aparecem como assimétricos. Isso pode afetar o tônus da MAP. Não é tão comum nos homens. **(** ***Novo*** **)**
      **2.6.2.1.6 Lesão da MAP:** por exemplo, lacuna do esfíncter anal palpável, embora em geral não seja comum, ao contrário das mulheres. **(** ***Novo*** **)**
      **2.6.2.2** **Função de contração da MAP:** aspectos para acessar
      **2.6.2.2.1 Contratilidade voluntária:** ^( 43 )^ o indivíduo é capaz de contrair a MAP sob demanda. Uma contração é sentida como uma ação de apertar, levantar e espremer sob/ao redor do dedo. **(** ***Novo*** **)**
      **2.6.2.2.2 Força:** ^( 43 )^ capacidade de geração de força de um músculo. Geralmente é expressa como contração voluntária máxima. **(** ***Novo*** **)**
      **2.6.2.2.3 Resistência:** ^( 43 )^ capacidade de sustentar a força quase máxima ou máxima, avaliada pelo tempo em que o paciente é capaz de sustentar uma contração estática ou isométrica máxima. **(** ***Novo*** **)**
      **2.6.2.2.4 Repetibilidade:** ^( 43 )^ capacidade de desenvolver repetidamente a força quase máxima ou máxima, determinada pela avaliação do número máximo de repetições que o paciente pode realizar antes do declínio detectável em vigor. Registre o número de contrações em uma linha. **(Novo)**
      **2.6.2.2.5** **Co-contração:** contração ou ativação de dois ou mais músculos ao mesmo tempo. Identifique quais músculos são cocontratores e se a cocontração é sinérgica. **(** ***Novo*** **)**
      **2.6.2.2.6 Capacidade de relaxamento:** retorno da MAP ao seu tônus de repouso original após a contração voluntária. Também inclui a capacidade de manter o relaxamento da MAP em antecipação a, ou durante, qualquer tipo de toque. **(** ***Novo*** **)**
      **2.6.2.3 Respos** **ta da MAP com o aumento da** **pressão intra-abdominal:** por exemplo, aspectos da tensão/Valsalva/tosse para avaliar.
      **2.6.2.3.1 Direção de contração** (elevação, descida)
  **2.6.3 Diagnósticos relacionados aos exames da MAP**
    **2.6.3.1 Músculos do AP hiperativos:** MAP que não relaxam ou podem até contrair quando o relaxamento é funcionalmente necessário – por exemplo, durante a micção ou a defecação. **(** ***Modificado*** **)**
    **2.6.3.2 Músculos do AP hipoativos:** músculos do AP que não podem contrair voluntariamente quando instruídos a fazê-lo ou se necessário. **(** ***Modificado*** **)**
**2.7 Diário miccional/gráfico de frequência-volume**
  **2.7.1 Gráfico de frequência-volume:** ^( 3 , 5 , 18 , 48 )^ o registro do horário de cada micção, juntamente do volume urinado (VU) por, pelo menos, 24 horas. Idealmente, um mínimo de três dias de registro (não necessariamente consecutivos) geralmente fornecerá dados clínicos mais úteis. É relevante discriminar a micção diurna e noturna.
  **2.7.2 Diário miccional:** adiciona ao gráfico de frequência-volume a ingestão de líquidos, o uso de eletrodos, os episódios de incontinência, o grau de incontinência e as circunstâncias no momento da perda. ** Sinais em que o gráfico de frequência-volume ou o diário miccional são importantes. Episódios de urgência e sensibilidade também podem ser registrados, assim como as atividades realizadas durante ou imediatamente antes da perda involuntária de urina. Informações adicionais obtidas do diário miccional envolvem gravidade da incontinência em termos de episódios de perda e uso de absorvente.
    **2.7.2.1 Período diurno:** ^( 18 )^ o período entre acordar com a intenção de se levantar até ir para a cama com a intenção de dormir (horas acordadas). **(** ***Novo*** **)**
    **2.7.2.2 Período noturno:** ^( 18 )^ o principal período diário de sono do indivíduo. Começa no momento de ir para a cama com a intenção de dormir e é concluído quando o indivíduo decide não mais tentar dormir e acordar para o dia seguinte. **(** ***Modificado*** **)**_NR 2.15, NR 2.16_
    **2.7.2.3 Período de sono principal:** ^( 18 )^ o período entre o momento de adormecer e o tempo de despertar para o dia seguinte.
    **2.7.2.4** **Noturno:** ocorrendo ou ativo à noite. ^( 1 )^ Por exemplo, sintomas e sinais que ocorrem à noite. ^(^^18^ **(** ***Modificado*** **)**
    **2.7.2.5 Fre** **quência (urinária) diurna:** ^( 3 , 5 )^ número de micções durante o dia (horas acordadas, incluindo primeira micção depois de acordar e última micção antes de dormir).**
    **2.7.2.6 Frequência (urinária) noturna:** ^( 18 )^ número total de micções noturnas, independentemente do sono.**
    **2.7.2.7 Noctúria:** número de vezes que um indivíduo urina durante o período de sono principal, desde o momento em que adormeceu até a intenção de se levantar desse período. Isso é obtido do diário miccional. ^( 18 )^
    **2.7.2.8 Frequência (urinária) de 24 horas:** ^( 3 , 5 , 18 , 48 )^ número total de micções diurnas e noturnas durante um período de 24 horas especificado.** **(** ***Modificado*** **)**
    **2.7.2.9 Volume de urina de 24 horas:** ^( 18 )^ somatória de todos os volumes de urina durante um período de 24 horas especificado. A primeira micção após acordar é descartada, e o período de 24 horas começa no momento da próxima micção, sendo completado com a inclusão da primeira micção, após levantar-se, no dia seguinte.** **(** ***Modificado*** **)**
    **2.7.2.10 Volume máximo urinado:** VU mais alto registrado durante o período de avaliação. **(** ***Modificado*** **)** Isso geralmente é igual a capacidade da bexiga.**
    **2.7.2.11 Volume médio urinado:** somatória de volumes urinados divididos pelo número de micções durante o período de avaliação.** **(** ***Modificado*** **)**
    **2.7.2.12 Média do volume máximo urinado (capacidade funcional):** volume médio máximo urinado em atividades diárias.**
    **2.7.2.13 Poliúria: produção excessiva de urina:** ^( 1 , 3 , 5 )^ foi definida como mais de 40mL de urina/kg de peso corporal durante 24 horas ou 2,8L de urina para um homem com 70kg. ^( 48 )^
    **2.7.2.14 Volume de urina noturno:** ^( 18 )^ volume total de urina produzida durante a noite. A medição de volume começa após a última micção antes do sono e termina após a primeira micção do dia (quando o indivíduo decide não mais tentar dormir). _NR 2.16_ **
    **2.7.2.15 Poliúria noturna:** ^(^^18 , 48^ ** maior produção proporcional de urina durante a noite em comparação com o volume de urina de 24 horas. _NR 2.17_ (Modificado). O índice de poliúria noturna é a definição mais comumente utilizada, sendo calculado da seguinte forma: volume urinário noturno/volume urinário de 24 horas × 100%.
- 33% em idosos, por exemplo,> 65 anos.
- >20% em indivíduos mais jovens.
- 20% a 33% na “meia-idade”.

**Figura 1:**
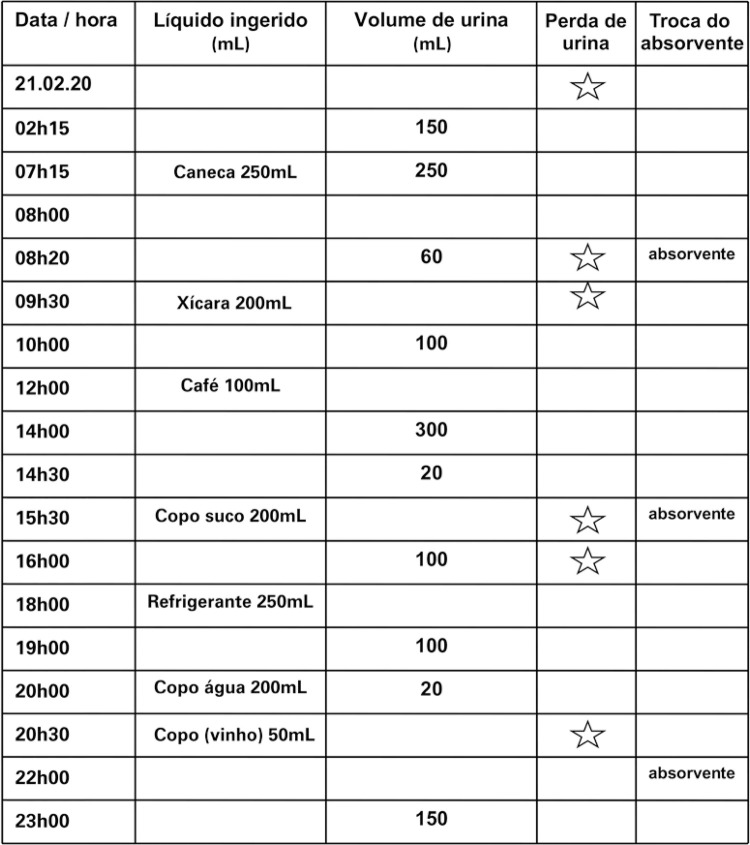
Diário miccional: Este gráfico simples permite que você registre o líquido que você bebe e a urina que elimina ao longo de 3 dias (não necessariamente consecutivos) na semana anterior à sua consulta. Isso pode fornecer informações valiosas. (i) Por favor, preencha aproximadamente quando e quanto líquido que você bebe e o tipo de líquido. (ii) Por favor, preencha o tempo e a quantidade (em mL) de urina eliminada e marque com uma estrela se tiver perda de urina ou marque com um “T” se você precisou trocar seu absorvente (veja abaixo um exemplo de como preencher este formulário). Frequência = 9; Noctúria = 1; Produção de urina / 24h = 1250mL; volume máximo urinado = 300mL; volume médio urinado = 125mL

A figura 1 fornece um exemplo de um diário miccional.

**2.7.2.16 Teste do absorvente:** para indivíduos com sintomas de incontinência urinária (ou fecal), a quantificação de urina (fezes) perdida ao longo da duração do teste pode ser medida pelo aumento no peso dos absorventes (peso pré e pós-teste) utilizados, o que pode fornecer um guia para a gravidade da incontinência. Durações diferentes de um teste curto (1 hora) a um teste de 24 e 48 horas foram usadas com provocação variando de atividades diárias normais a regimes definidos. _NR 2.18_

## Notas de rodapé para a seção 2

2.1: Há poucas evidências em ensaios clínicos de que a realização de um exame clínico melhore os cuidados, mas o consenso geral sugere que ele continua sendo parte essencial da avaliação de homens com incontinência urinária ^( 2 )^ ou outros STUI.

2.2: Uma bexiga normal no adulto não pode ser palpada ou percutida até que haja pelo menos um volume de 150mL de urina. Em volumes maiores, de cerca de ≥500mL, uma bexiga distendida pode ser visível em pacientes magros como uma massa abdominal inferior da linha média. A percussão é melhor que a palpação para diagnosticar uma bexiga distendida. O examinador começa percutindo um pouco acima da sínfise púbica e continua cefálico, até que haja uma mudança no tom de obtuso para ressonante. ^( 30 )^

2.3: Se a fimose for grave, pode causar sintomas miccionais. A maioria dos cânceres penianos ocorre em homens não circuncisados e surge no prepúcio ou glande, podendo estar associada a sintomas miccionais. ^( 33 )^

2.4: Anormalidades escrotais podem ajudar a elucidar os sintomas do TUI em homens. Por exemplo: homens com epididimite podem ter sintomas de infecção urinária associados à bacteriúria por coliformes. ^( 34 )^

2.5: A orquite isolada secundária à ITU é rara, no entanto, a infecção por micobactérias, a caxumba e o tratamento com Bacilo de Calmette Guérin podem causar orquite. ^( 34 )^

2.6: Se muito grandes, podem distorcer o escroto e a uretra e interferir na micção normal. Uma hidrocele é, por vezes, secundária a tumor testicular ou processos inflamatórios no epidídimo ou orquite.

2.7: A presença de hérnias, inchaço cístico no escroto e tumores testiculares deve ser excluída por exame clínico cuidadoso.

2.8: Durante o toque retal, o tamanho da próstata e a consistência podem ser estimados, embora o toque retal tende a subestimar o tamanho real da próstata. ^( 35 , 36 )^

2.9: Em pacientes com fístulas reto-uretrais, esta pode ocasionalmente ser palpada na parede anterior do reto. O local da fístula no esfíncter anal ou acima dele pode ocasionalmente ser observado junto do grau de endurecimento da parede anterior do reto. Com grandes fístulas, a uretra pode ser palpada, especialmente se houver um cateter de Foley no lugar.

2.10: Por exemplo, uma pessoa com Parkinson pode ser incapaz de realizar cateterismo intermitente por causa do tremor. Um exame neurológico focado também é recomendado, especialmente em pacientes com suspeita de disfunção neurogênica da bexiga. ^( 38 )^ A diminuição da sensibilidade perineal e do tônus do esfíncter anal pode ser sinal de neuropatia. ^( 41 )^

2.11: Esse reflexo é mais comumente testado colocando-se um dedo no reto e, depois, apertando-se a glande. Se um cateter de Foley estiver no lugar, o reflexo bulbo-esponjoso também pode ser estimulado, puxando gentilmente o cateter. Se o reflexo bulbo-esponjoso estiver intacto, o aperto do esfíncter anal deve ser sentido e/ou observado. ^( 39 )^ O reflexo bulbo-esponjoso testa a integridade do arco reflexo mediado pela medula espinhal, envolvendo S2-S4, e pode estar ausente na presença de anormalidades da medula sacral ou do nervo periférico. ^( 39 )^

2.12: Se o paciente teve cirurgia ou trauma na uretra ou na bexiga, o examinador deve verificar se a perda urinária ocorre por meio de uma fístula na cicatriz ou em qualquer outro local do pênis, períneo, virilha ou parte inferior do abdômen.

2.13: Normalmente, há movimento interno (cefálico) do períneo e do ânus.

2.14: Isso tudo faz parte da realização do toque retal, avaliando esfíncteres anais em puborretal.

2.15: Para fins de terminologia de noctúria, a noite é, portanto, definida pelo ciclo de sono do indivíduo, ao invés do ciclo solar (do pôr do sol ao nascer do sol). Assim, alguns trabalhadores em turnos podem ter seu período de “noite” durante as horas do dia, pois é a hora do seu período de sono principal. ^( 18 )^

2.16: A medição do volume começa após a última micção antes do sono e termina após a primeira micção do dia. A primeira micção diurna segue a decisão do indivíduo de que não mais tentará dormir. ^( 18 )^

2.17: Existem várias definições na literatura que podem ser usadas para indicar poliúria noturna, incluindo: ^( 18 )^

Produção noturna de urina baseada no peso maior que 10mL/kg. ^( 49 )^

1. Taxa de produção de urina noturna >90mL/h, ^( 50 )^ sugestiva de poliúria noturna em homens (cerca de 450mL por 8 horas de sono). ^( 51 )^ Não há estudos sobre a taxa de produção noturna de urina em mulheres, que pode ser diferente da dos homens.
2. O índice de poliúria noturna é a definição mais comumente usada para poliúria noturna ^( 52 )^ (volume noturno de urina/volume de 24 horas) com base no volume noturno de urina como parte do volume total de urina de 24 horas (idade dependente).
3. Índice de noctúria (volume noturno de urina/volume máximo urinado) ^( 53 )^ >1: a noctúria ocorre porque o volume máximo urinado é menor que o de urina noturno; > 1,5: noctúria secundária à superprodução noturna de urina em excesso da capacidade máxima da bexiga, isto é, poliúria noturna.

2.18: Um teste da fralda quantifica a gravidade da incontinência e pode ser a medida mais objetiva da incontinência. A gravidade da incontinência (quantificada pelo peso da fralda) afeta os resultados da cirurgia. O teste de 24 horas e o diário miccional são instrumentos confiáveis para avaliar o grau de perda urinária e o número de episódios de incontinência, respectivamente. Aumentar a duração do teste para 48 e 72 horas aumenta a confiabilidade, mas está associado à diminuição da adesão do paciente. ^( 54 )^ No geral, o teste domiciliar de 24 horas é o mais acurado para quantificação e diagnóstico de incontinência urinária, porque é o mais reprodutível. ^( 55 )^ O teste da fralda de 1 hora pode ser usado porque é fácil e padronizado, no entanto, não há paralelo estrito com o teste de 24 horas e pode subestimar a fraqueza do esfíncter na parte posterior do dia.

## SEÇÃO 3 – INVESTIGAÇÕES URODINÂMICAS

**Urodinâmica:** medição de todos os parâmetros fisiológicos relevantes da função e de qualquer disfunção do TUI. ^( 56 , 57 )^_NR 3.1, NR 3.2_ **( *Novo* )**

**Sequência clínica dos exames:** ^( 3 , 5 )^ a avaliação urodinâmica geralmente envolve um indivíduo que chega com bexiga confortavelmente cheia para urofloxometria livre (sem cateter) e medida do RPM, antes da cistometria e do estudo de pressão/fluxo. **( *Novo* )**

**3.1 Urofloxometria**
  **3.1.1 Condições ideais para urofluxometria livre (sem cateter):** todo o estudo de urofluxometria livre deve ser realizado de forma totalmente privada, em sala de urofluxometria. A maioria dos urofluxômetros modernos tem alto grau de precisão (+/− 5%), embora a calibração regular seja importante ( Figura 2 ). ^( 58 )^
    **Figura 2:**
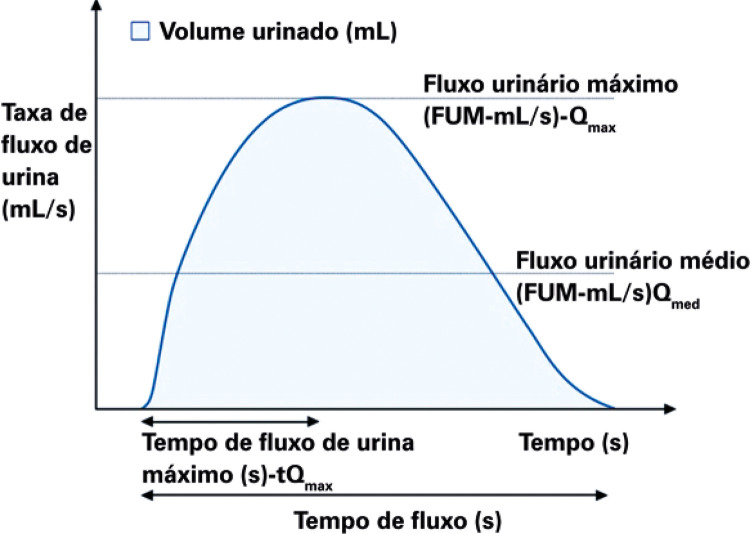
Representação esquemática do fluxo de urina ao longo do tempo e parâmetros da urofluxometria
  **3.1.2 Fluxo urinário:** passagem uretral da urina, na qual o padrão do fluxo de urina pode ser: ^( 2 , 3 , 5 )^
    **3.1.2.1 Contínuo:** sem interrupção do fluxo de urina.
    **3.1.2.2 Intermitente:** o fluxo de urina é interrompido.
  **3.1.3 Taxa de fluxo da urina (TFU; unidade: mL/s):** volume de urina expelido pela uretra por unidade de tempo. ^( 2 , 3 , 5 )^
  **3.1.4** **Volume urinado (unidade: mL):** volume total de urina expelido pela uretra durante uma única micção. ^( 2 , 3 , 5 )^ **(** ***Modificado*** **)**
  **3.1.5 Taxa de fluxo máximo (urina) (TFMax; unidade: mL/s) – Q**_max_ **:** valor máximo medido da taxa de fluxo urinário ^( 2 , 3 , 5 )^ corrigido de artefatos. ^( 3 , 5 )^
  **3.1.6 Tempo de fluxo (TF; unidade: segundos):** tempo durante o qual o fluxo mensurável realmente ocorre. ^( 2 , 3 , 5 )^
  **3.1.7** **Taxa de fluxo médio (urina) (TFMed; unidade:** **mL/s) – Q**_ave_ **:** VU dividido pelo TF. ^( 2 , 3 , 5 )^
  **3.1.8 Tempo de micção (TM; unidade: segundos):** duração total de micção, incluindo interrupções. Quando a micção é completada sem interrupção, o TM é igual ao TF. ^( 2 , 3 , 5 )^
  **3.1.9 Tempo até a taxa máxima de fluxo urinário (tQ**_**max**_ **; unidade: segundos):** tempo decorrido desde o início do fluxo de urina para o fluxo máximo de urina. ^( 2 , 3 , 5 )^
  **3.1.10 Interpretação da normalidade da urofluxometria livre:** por causa da forte dependência dos valores de fluxo urinário em homens no VU ^( 59 , 60 )^ e idade, ^( 60 )^ estes são melhor referenciados em nomogramas, ^( 60 - 63 )^ nos quais o valor da normalidade foi determinado e validado. O indivíduo deve comentar se a micção foi representativa de seu fluxo urinário usual e se ele tem variação diurna no fluxo urinário ( Figuras 3A e B ). **(** ***Novo*** **)**
    **Figura 3:**
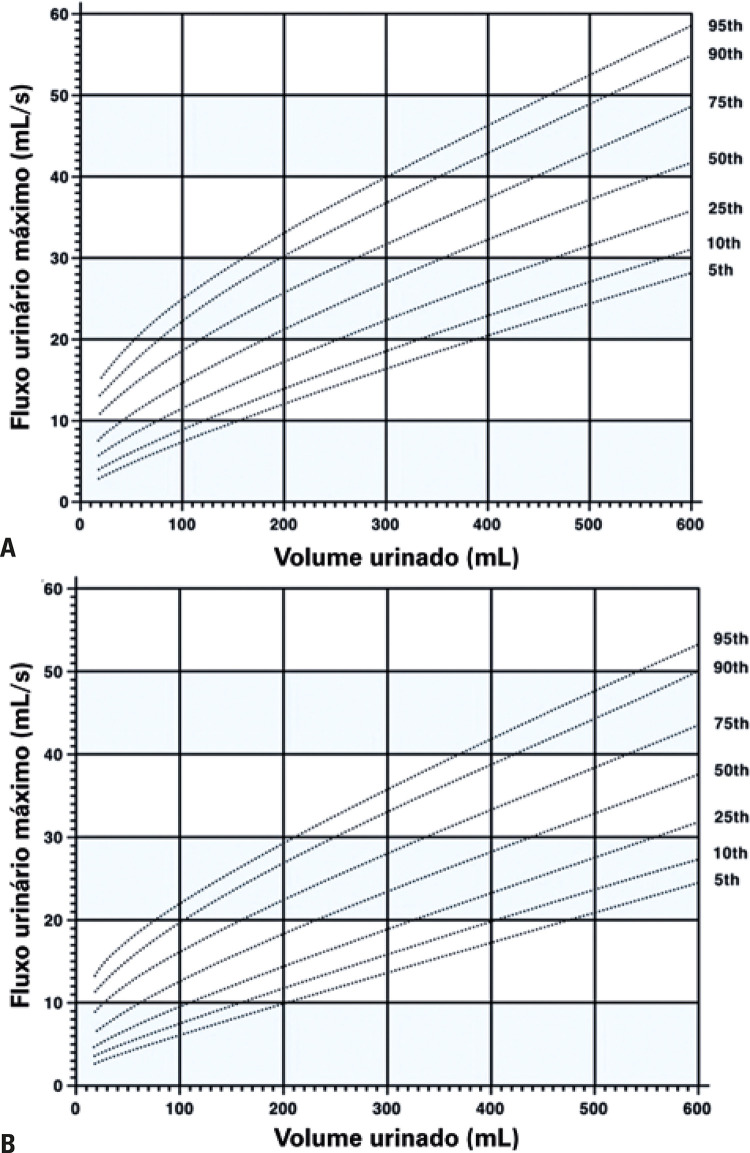
A e B, mostram os nomogramas de Liverpool (60) para a taxa máxima de fluxo de urina em homens (i) até 50 anos (média de 35 anos) e (ii) mais de 50 anos (média de 60 anos). A equação para o nomograma da taxa de fluxo máximo de urina (dividido pela idade como acima) é: Raiz quadrada (Fluxo urinário máximo) = 2,37 + 0,18 Raiz quadrada (volume urinado) - 0,014 x idade. (Raiz do erro quadrático médio: 0,727) ( *Novo* ). Referências a um específico taxa de fluxo de urina como o limite inferior do normal, desde que um volume específico tenha sido urinado, requerem estudos de validação

O 25º percentil pareceu ser mais adequado para limites inferiores de normalidade para ambas as taxas de fluxo para identificar aqueles homens mais propensos a ter disfunção miccional (mais comumente OIV). ^( 64 )^ Altos percentis de taxa de fluxo urinário ocorrem em homens com hiperatividade detrusora. ^( 64 )^_NR 3.3_ Algumas diferenças raciais em taxas de fluxo de urina foram relatadas. _NR 3.4_ Idealmente, estudos de urofluxometria alterados devem ser repetidos. **( *Novo* )**

**3.2 Resíduo pós-miccional (volume de urina; unidade: mL):** volume de urina na bexiga depois do fim da micção. ^( 2 , 3 , 5 )^
  **3.2.1 Condições para medição do RPM:** o RPM elevado é erroneamente medido por atraso na aferição devido a débito renal adicional (1mL/minuto a 14mL/minuto) na bexiga. _NR 3.5_ O ultrassom permite registro imediato (dentro de 60 segundos após a micção) e minimiza esse erro. A inserção imediata de cateter transuretral para drenagem da bexiga ainda pode fornecer uma medição do RPM eficaz e precisa. Os cateteres uretrais, no entanto, podem não ter eficácia de drenagem igual. _NR 3.6_ A medição do RPM pelo ultrassom idealmente deve ser repetida em pelo menos uma vez, se o RPM estiver elevado. **(** ***Novo*** **)** Bexiga hiperdistendida, ao invés de “confortavelmente cheia”, pode levar a RPM falsamente elevado, avaliado posteriormente por repetição da micção/repetição na medida do RPM.
  **3.2.2 Avaliação da normalidade da RPM:** limite superior normal em homens sem LUTS é dependente da idade, com estudos relatando valor de corte de 10mL a 30mL. ^( 67 - 70 )^ Não há dados adequados atualmente disponíveis a partir dos intervalos esperados/característicos de RPM em homens com sintomas do trato inferior disfunção. Tais estudos precisariam refletir a precisão da medição, incluindo se a medição de RPM é “imediata” (por exemplo: por ultrassom) ou por cateterismo uretral (a menos que seja também “imediato”). Na falta desses estudos, nossa opinião consensual é a de que RPM (ultrassom) acima de 50mL, seguido de dupla micção, pode levar à suspeita de disfunção miccional. **(** ***Novo*** **)**
**3.3 Cisto** **metri** **a geral** ^( 2 , 3 , 5 , 56 , 57 )^
  **3.3.1 Estudo urodinâmico:** geralmente ocorre em uma sala especial (laboratório urodinâmico) e envolve enchimento da bexiga (artificial) com líquido especificado (a ICS recomenda solução fisiológica salina ou solução de contraste de raios X, se estudos com vídeo) e em velocidade de infusão igualmente especificada. ^( 2 , 3 , 56 , 57 )^_NR 3.7_
  **3.3.2 Cistometria:** medição da relação pressão/volume da bexiga durante o enchimento. ^( 2 , 3 , 56 , 57 )^_NR 3.7_
  **3.3.3 Cistometrograma:** gravação gráfica das pressões e dos volumes da bexiga ao longo do tempo. ^( 2 , 3 , 56 , 57 )^
  **3.3.4 Condições para cistometria incluindo**
    3.3.4.1 Pressões (zerar).*
    3.3.4.2 Transdutores de pressão.*
    3.3.4.3 Transdutores montados em cateter.*
    **3.3.4.4 Volume inicial da bexiga.**
    3.3.4.5 Líquido*_NR 3.7_ * Abordado em referências ^( 56 , 57 )^
    **3.3.4.6 Temperatura do líquido:** o líquido é geralmente utilizado na temperatura da sala. Pode ser aquecido à temperatura do corpo, mas sem evidência de que isso influencie nos resultados. ^( 71 , 72 )^_NR 3.8_ **(** ***Modificado*** **)**
    **3.3.4.7 Posição do paciente:** posição sentada (em pé) é mais provocativa para alteração da atividade detrusora (isto é, hiperatividade) do que a posição supina. Em algum ponto no teste, o enchimento pode desejavelmente ocorrer com o paciente em pé (naqueles pacientes capazes de fazê-lo). ^( 71 , 73 )^_NR 3.9_ **(** ***Modificado*** **)** Muitos homens urinam em pé.
    **3.3.4.8 Velocidade de enchimento:** a velocidade de enchimento, incluindo quaisquer alterações durante o teste, deve ser anotada no relatório urodinâmico. ^( 3 , 56 , 57 , 71 , 73 - 76 )^_NR 3.10_ A velocidade média de enchimento (25mL/minuto a 50mL/minuto) deve ser utilizada na maioria dos estudos de rotina. A velocidade de enchimento muito mais lenta (25mL/minuto) é apropriada em homens para os quais há preocupação com baixa complacência ou diário miccional mostrando baixa capacidade vesical, ou aqueles com bexiga neuropática. Maior velocidade de enchimento é superior a 50mL/minuto. **(** ***Modificado*** **)**
  **3.3.5** **Pressão intravesical (P**_ves_ **; unidade: cmH**_2_ **O):** a pressão dentro da bexiga (diretamente medida pelo cateter intravesical). ^( 2 , 3 , 56 , 57 )^
  **3.3.6 Pressão abdominal (P**_abd_ **; unidade: cmH2O):** a pressão na cavidade abdominal em torno a bexiga. É geralmente estimada a partir de medição da pressão retal, embora a pressão por meio de estoma intestinal possa ser medida alternativamente. _NR 3.11_ A medição simultânea da P _abd_ é essencial para a interpretação do traçado da P _ves_ . ^( 2 , 3 , 5 )^ Artefatos no traçado da pressão do detrusor (P _det_ ) podem ser produzidos por contração retal. ^( 2 , 3 , 56 , 57 )^ ( ***Modificado*** )
  **3.3.7 Pressão do detrusor (P**_**det**_ **; unidade: cmH**_2_ **O):** o componente da P _ves_ que é criado por forças na parede da bexiga (passivo e ativo). É calculado subtraindo a P _abd_ da P _ves_ (P _det_ = P _ves_ –P _abd_ ). ^( 2 , 3 , 56 , 57 )^_NR 3.12_
**3.4 Cistometria** ^( 2 , 3 , 56 , 57 )^
  **3.4.1 Cistometria:** relação pressão/volume da bexiga durante seu enchimento. Começa com o início do enchimento e termina quando uma “permissão para urinar” é dada pelo urodinamicista ^( 2 , 3 , 5 , 56 , 57 )^ ou com incontinência (perda involuntária) do conteúdo da bexiga ( Figura 4 ). ^( 71 )^ **(** ***Modificado*** **)**
    **Figura 4:**
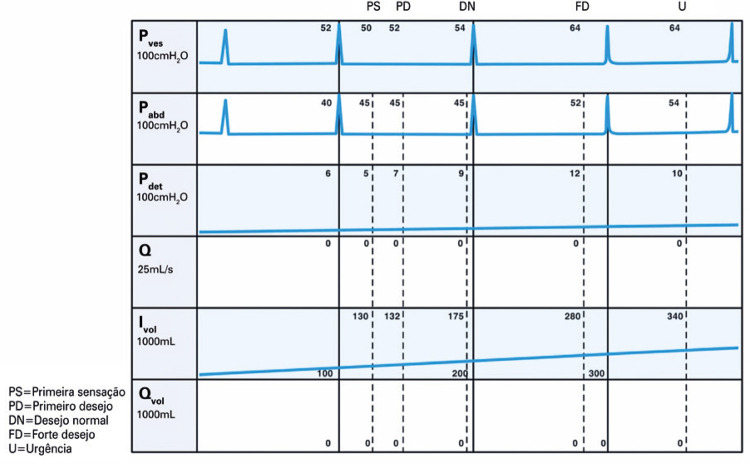
Cistometria normal na urodinâmica multicanal. Primeiro desejo: 132mL; desejo normal: 175mL; forte desejo para urinar: 280mL; urgência: 340mL. A contração do detrusor está ausente durante a cistometria. São demonstrados artefatos de tosse e boa subtração de pressão abdominal de pressão intravesical para obter pressão do detrusor

P _ves_: pressão intravesical; P _abd_: pressão abdominal; P _det_: pressão do detrusor; Q: fluxo urinário; I _vol_: volume infundido; Q _vol_: volume urinado.
  **3.4.2 Objetivos da cistometria:** avaliar a sensação da bexiga, a capacidade vesical, a atividade detrusora e a complacência, bem como documentar (a situação e P _det_ durante) a perda urinária. **(** ***Modificado*** **)**
  **3.4.3 Sensação da bexiga durante a cistometria:** geralmente avaliada pelo questionamento do indivíduo em relação à plenitude da bexiga durante a cistometria.
    **3.4.3.1 Primeira sensação de enchimento da bexiga:** a sensação quando o indivíduo sente pela primeira vez o enchimento da bexiga. ^( 3 , 5 , 71 , 75 )^_NR 3.13_
    **3.4.3.2 Primeiro desejo de urinar:** a primeira sensação que o indivíduo tem de urinar. ^( 3 , 5 )^_NR 3.13_
    **3.4.3.3 Desejo normal de urinar:** a sensação que leva o indivíduo a urinar no próximo momento conveniente, mas pode ser adiada, se necessário. ^( 3 , 5 )^
    **3.4.3.4 Forte desejo de urinar:** o persistente desejo de urinar sem o medo de perda de urina. ^( 3 , 5 , 71 )^_NR 3.13_
    **3.4.3.5 Urgência:** desejo repentino e irresistível para urinar, o qual é difícil retardar. _NR1.4, NR1.5_
    **3.4.3.6 Hipersensibilidade vesical**: ^( 5 )^ aumento da sensibilidade da bexiga durante o enchimento como: **(** ***Novo - masculino*** **)**
- Primeiro desejo para urinar precoce.
- Desejo forte de anular, que ocorre em volume pequeno da bexiga.
- Capacidade cistométrica máxima pequena **(3.4.4.2).**
- Sem aumento anormal na P _det_ .
  **3.4.3.7 Sensibilidade vesical diminuída:** sensação da bexiga percebida como diminuída durante a cistometria.
  **3.4.3.8 Sensibilidade vesical ausente:** ausência de sensação da bexiga durante a cistometria, pelo menos até a capacidade esperada de 500mL.
  **3.4.3.9 Dor:** queixa de dor durante a cistometria é anormal. Seu local, caráter e duração devem ser anotados.
  **3.4.4 Capacidade da bexiga durante a cistometria** ^( 3 , 5 , 56 , 57 )^
  **3.4.4.1 Capacidade cistométrica (unidades: mL):** volume da bexiga no final do enchimento vesical, quando a “permissão para urinar” é geralmente dada pelo urodinamicista. Esse ponto final e o nível de sensação de bexiga do indivíduo, por exemplo, “desejo normal de urinar”, devem ser anotados. Esse ponto final pode ser maior do que o normal em homens com sensação da bexiga reduzida.
  **3.4.4.2 Capacidade cistométrica máxima (unidade: mL):** em indivíduos com sensação normal, esse é o volume quando não se pode mais retardar a micção durante o enchimento vesical. _NR 3.14, NR 3.15, NR 3.16_
**3.4.5 Função detrusora durante o enchimento vesical**
  **3.4.5.1 Atividade/função do detrusor normal:** ^( 3 , 5 )^ há pouca ou nenhuma mudança na pressão durante o enchimento. Não há contrações detrusoras espontâneas e nem provocadas por atividades como mudanças posturais, tossir ou ouvir o som de água corrente. _NR 3.17_ **(** ***Modificado*** **)**
  **3.4.5.2 Hiperatividade do detrusor (HD):** ^( 3 , 5 )^ ocorrência de contrações do detrusor durante o enchimento vesical. Essas contrações, que podem ser espontâneas ou provocadas, produzem uma forma de onda no cistometrograma, de duração variável e amplitude. As contrações podem ser fásicas ou terminais. Elas podem ser suprimidas pelo paciente ou incontroláveis. **(** ***Modificado*** **)** Sintomas como, por exemplo, urgência e/ou incontinência de urgência ou percepção da contração podem ou não ocorrer (anote se presentes).
  **3.4.5.2.1 HD (primária) idiopática:** contração do detrusor de causa não identificável ( Figura 5 ). **(** ***Modificado*** **)**
    **Figura 5:**
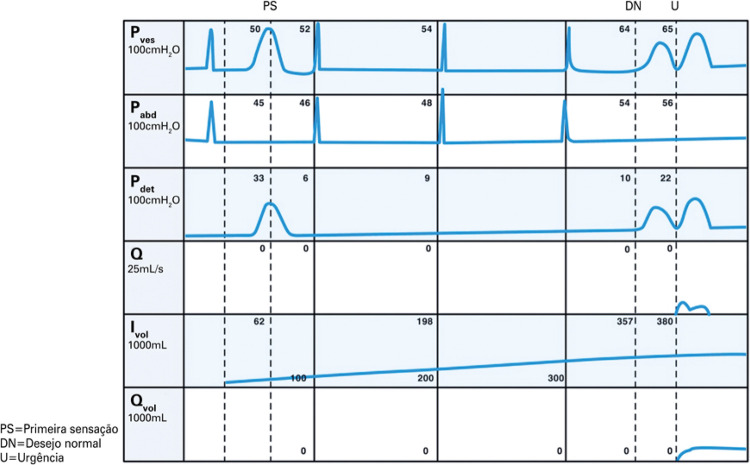
Cistometria demonstrando hiperatividade do detrusor: o primeiro desejo miccional ocorreu com 62mL junto com contração do detrusor; desejo normal de urinar com 357mL; urgência com 390mL seguida por uma contração do detrusor. Também há alta pressão e fluxo baixo durante a micção

P _ves_: pressão intravesical; P _abd_: pressão abdominal; P _det_: pressão do detrusor; Q: fluxo urinário; I _vol_: volume infundido; Q _vol_: volume urinado.
  **3.4.5.2.2 HD (secundária) neurogênica:** ^( 3 , 5 , 13 )^ HD e evidência (história; *deficit* visível ou mensurável) de alteração neurológica. **(** ***Modificado*** **)**
  **3.4.5.2.3 HD (secundária) não neurogênica:** uma possível causa identificável não neurológica existe para contração involuntária do detrusor durante o enchimento da bexiga. Por exemplo: funcional (obstrução), cálculo, tumor (por exemplo: carcinoma *in situ* ), ITU. _NR 3.18_ **(** ***Modificado*** **)**
  **3.4.6 Complacência vesical (detrusora) (unidade: mL/cm H**_2_ **O)** ^( 3 , 5 , 56 , 57 , 77 - 79 )^
    **3.4.6.1 Descrição:** relação entre a mudança no volume da bexiga e mudança simultânea na P _det_ como uma medida da distensibilidade da bexiga. ^( 3 , 5 )^
    **3.4.6.2 Cálculo:** divisão da diferença de volume (ΔV) pela diferença na P _det_ (ΔP _det_ ) simultânea durante a cistometria (C=ΔV/ΔP _det_ ). A complacência reflete a quantidade de líquido na bexiga necessária para aumentar a pressão da bexiga em 1cmH _2_ O e é expressa em mL por cmH _2_ O.
    **3.4.6.3.** Fatores que afetam a medição de complacência da bexiga:
    **3.4.6.3.1 Velocidade de enchimento da bexiga:** bexiga deve ser enchida em até 50mL/minuto, se não houver razão para suspeitar de bexiga de baixa complacência. Enchimento mais rápido é mais provocativo e pode reduzir artificialmente a complacência da bexiga. Esse artefato pode ser resolvido quando o enchimento é interrompido ou repetido com velocidade mais lenta. **(** ***Modificado*** **)**
    **3.4.6.3.2 Propriedades contráteis/relaxantes do detrusor (complacência diminuída):** propriedades da parede da bexiga que podem reduzir a complacência. Por exemplo: radiação pélvica, quimioterapia ou bexiga hiperdistendida. **(** ***Modificado*** **)** A OIV pode resultar em hipertrofia do músculo detrusor e deposição de colágeno intramural e de elastina, além de contribuir para a complacência reduzida. **(** ***Novo*** **)**
    **3.4.6.3.3 Outros fatores que afetam a complacência vesical** ^( 78 )^ (aumento complacência): divertículo da bexiga (também pseudodivertículo) e refluxo vesicoureteral (alto grau). **(** ***Novo*** **)**
    **3.4.6.4 Ponto inicial para cálculo da complacência:** geralmente a P _det_ no início do enchimento da bexiga e o volume da bexiga correspondente (geralmente zero). ^( 3 )^ Atenção especial deve ser dada para garantir que a bexiga seja esvaziada no início da medição; o esvaziamento incompleto pode diminuir artificialmente a complacência da bexiga. **(** ***Modificado*** **)**
    **3.4.6.5 Ponto final para cálculos de complacência:** pressão de detrusor (e correspondente volume da bexiga) na capacidade cistométrica (tempo para a pressão se estabilizar após a parada do enchimento). Ambos os pontos são medidos excluindo a contração do detrusor. No caso de HD com perda, ambos os pontos devem ser medidos imediatamente antes do início da contração do detrusor (e, portanto, faz com que o volume da bexiga diminua, afetando os cálculos de complacência). Baixa complacência foi definida (em mulheres) como complacência da bexiga <10mL/cmH _2_ O (neurogênica) ou <30mL/cmH _2_ O (não neurogênica). A complacência normal é >30mL/cmH _2_ O (neurogênica) e 40mL/cmH _2_ O (não neurogênica). ^( 79 )^ Valores recomendados no homem ainda não estão bem definidos. _NR 3.19_ ***(Modificado*** **)**
  **3.4.7 Repetição da cistometria:**_NR 3.20_ a repetição da urodinâmica, em caso de função vesical alterada, discrepâncias entre história e achados urodinâmicos suspeitos, erros técnicos e/ou artefatos, quando observados em análise imediata pós-teste. **(** ***Modificado*** **)**
  **3.4.8 Teste urodinâmico padrão ICS:** ^( 57 )^ urofluxometria, RPM, cistometria e estudo pressão/fluxo são termos do teste urodinâmico padrão ICS (ICS-SUT). _NR 3.21_ **(** ***Modificado*** **)**
**3.5 Função uretral durante a cistometria (uretrocistometria):** como a uretrocistometria é menos explorada nos homens do que nas mulheres, os leitores são direcionados para outras publicações sobre a metodologia. ^( 56 , 57 , 80 )^
**3.6 Mecanismo de fechamento uretral**
  **3.6.1 Mecanismo de fechamento uretral normal:** a pressão positiva de fechamento uretral é mantida durante o enchimento da bexiga, mesmo na presença de aumento da P _abd_ , embora possa ser superado pela HD.
  **3.6.2 Mecanismo de fechamento uretral incompetente:** a perda de urina ocorre durante atividades que podem elevar a pressão intra-abdominal, na ausência de contração do detrusor.
    **3.6.2.1 Incontinência de esforço urodinâmica:** perda de urina involuntária durante a cistometria, associada com aumento da pressão intra-abdominal, na ausência de contração detrusor.
    **3.6.2.2 Subtipo:** deficiência esfincteriana intrínseca : mecanismo de fechamento uretral muito enfraquecido.
    **3.6.3 Pressões de perda:** ^( 2 , 3 , 5 , 80 - 82 )^ existem dois tipos de medição da pressão de perda. Os valores de pressão de perda devem ser medidos no momento da perda.
      **3.6.3.1 Pressão de perda do detrusor (unidade: cmH**_2_ **O):** esse é um teste estático. A pressão é o valor mais baixo da P _det_ em que a perda de urina é observada durante a cistometria na ausência de aumento da P _abd_ . A pressão de perda do detrusor é o reflexo da resistência da saída da bexiga ou esfíncter uretral. Pressão de perda do detrusor alta (por exemplo: mais de 40cmH _2_ O) ^( 80 )^ pode colocar os pacientes em risco de deterioração do trato urinário superior, ou dano secundário à bexiga nos casos de doença ou lesão neurológica, como lesão medular ou esclerose múltipla. ^( 81 )^ Não existem dados sobre qualquer correlação entre pressão de perda do detrusor e dano do trato superior em pacientes não neurogênicos. **(** ***Modificado*** **)**
      **3.6.3.2 Pressão de perda sob esforço abdominal (unidade: cmH**_2_ **O):** esse é um teste dinâmico. A P _abd_ é intencionalmente aumentada, o que provoca perda de urina na ausência de contração do detrusor. ^( 82 )^ O paciente pode conseguir por meio de tosse ou prensa (pressão de perda com manobra de Valsava). A pressão de perda com manobra de Valsava permite medir a mais baixa pressão (medida pela pressão vesical ou P _abd_ ) que causa perda urinária.
  **3.7 Estudo pressão/fluxo** ^( 2 , 3 , 5 , 56 , 57 )^_NR 3.24_
    **3.7.1 Estudo pressão/fluxo:** relação pressão/volume (fluxo urinário) da bexiga durante a micção. ^( 1 - 3 , 5 , 56 , 57 )^ Começa quando a “permissão para urinar” é dada pelo urodinamicista e termina quando o homem considera que sua micção terminou. As medições a serem registradas devem ser a P _ves_ e a P _abd_ e calcular a (P _det_ , bem como a TFU. _NR 3.22_ **(** ***Modificado*** **)**
    **3.7.2 P**_**det**_ **e outras medições durante estudo de pressão/fluxo** ( Figura 6 ) ^( 2 , 3 , 5 , 56 , 57 )^
      **Figura 6:**
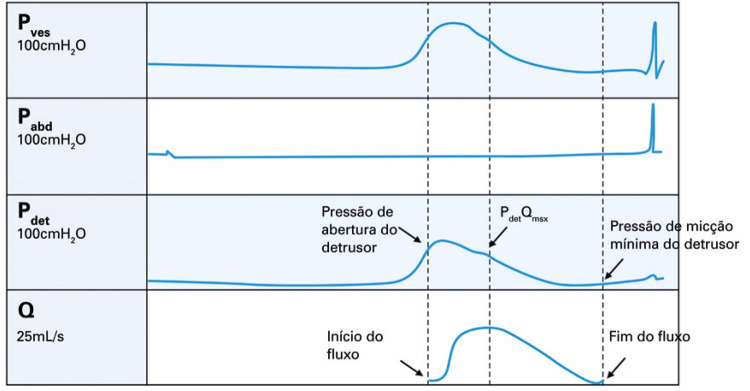
Diagrama esquemático de estudo de pressão fluxo e parâmetros de pressão e de fluxo

P _ves_: pressão intravesical; P _abd_: pressão abdominal; P _det_: pressão do detrusor; Q: fluxo urinário.
    **3.7.2.1 Pressão de abertura do detrusor (unidade: cmH**_2_ **O):** P _det_ registrada imediatamente antes do começo de fluxo de urina. **(** ***Modificado*** **)**
    **3.7.2.2 Atraso do fluxo (unidade: segundos):** o tempo decorrido desde o aumento inicial da pressão até o início do fluxo. Esse é o período inicial da contração isovolumétrica da micção. Isso reflete o tempo necessário para o líquido passar do ponto de medição da pressão para o transdutor de fluxo. _NR 3.23_ **(** ***Modificado*** **)**
    **3.7.2.3 P**_**det**_ **na abertura uretral (unidade: cmH**_2_ **O):** P _det_ registrada no início da medição do fluxo (considere o tempo de atraso; geralmente é de menos de 1 segundo).
    **3.7.2.4 Pressão do detrusor máxima (unidade: cmH**_2_ **O):** máxima P _det_ registrada durante a micção.
    **3.7.2.5 Pressão do detrusor no fluxo máximo (P**_**det**_ **.Q**_**max**_ **; unidade: cmH**_2_ **O):** P _det_ registrada no fluxo urinário máximo.
    **3.7.2.6 Pressão do detrusor no final do fluxo (unidade: cmH**_2_ **O):** P _det_ registrada no final do fluxo de urina.
    **3.7.2.7** **Contração do detrusor pós-miccional:** um aumento na P _det_ após a cessação do fluxo de urina. **(** ***Novo*** **)**
**3.7.3 Função detrusora durante a micção** ^( 2 , 3 , 5 , 56 , 57 )^
  **3.7.3.1 Função contrátil do detrusor normal:** a micção normal em homens é alcançada por uma contração do detrusor contínua, que leva ao esvaziamento completo da bexiga dentro de um intervalo de tempo normal. Depende da iniciação e da estimulação central dos reflexos envolvido. A amplitude da contração do detrusor (força/potência de contração do detrusor) tende a aumentar em resposta a qualquer aumento da resistência da uretra até a bexiga estar vazia. ^( 83 )^ **(** ***Modificado*** **)**
  **3.7.3.2 Detrusor hipoativo (DH):** baixa P _det_ ou tempo de contração do detrusor pequeno, geralmente em combinação com baixa TFU, resultando no esvaziamento prolongado da bexiga e/ou na falha em alcançar esvaziamento completo da bexiga dentro de um intervalo de tempo normal (observação: o termo “detrusor hipocontrátil”, ou hipocontratilidade do detrusor, descreve uma contração detrusora de força reduzida). A HAD pode ser de causa neurogênica ^( 13 , 84 )^ ou não neurogênica. **(** ***Modificado*** **)**
  **3.7.3.3 Detrusor acontrátil:** não é registrada contração do detrusor (ou seja, ausência de aumento da P _det_ ) durante o estudo urodinâmico, resultando em falha da micção. **(** ***Modificado*** **)** Micção parcial pode ocorrer por prensa. A possibilidade de “inibição” de contração do detrusor na micção deve ser considerada se o homem conseguir realizar a micção normalmente após a cistometria. Detrusor acontrátil pode ser de causa neurogênica ou não neurogênica. Detrusor acontrátil neurogênico deve substituir o termo “arreflexia do detrusor”.
**3.8 Função uretral durante a micção:** pode ser interpretada pelo registro pressão/fluxo e monitorado pela videocistouretrografia (videourodinâmica, **item** **4.3.4** ) e EMG ( **item** **3.9** ) quando disponível.
  **3.8.1 Função uretral normal durante a micção:** início da micção começa com relaxamento voluntário do AP e do esfíncter estriado (rabdoesfíncter). A bexiga, então, contrai-se com seu colo, que se abre, devido ao seu arranjo espiral de fibras. A micção é iniciada, sendo a uretra continuamente relaxada, para permitir a micção sob P _det_ e fluxo de urina normais, resultando em esvaziamento completo da bexiga. ^( 85 , 86 )^_NR 3.24, NR 3.25, NR 3.26_
  **3.8.2 Função uretral anormal durante a micção:** os esfíncteres uretrais não relaxam completamente ou eles são (temporariamente) contraídos durante a micção, resultando em aumento da P _det_ . O esvaziamento da bexiga pode estar completo ou incompleto (RPM presente).
    **3.8.2.1 Obstrução infravesical:** ^( 87 , 88 )^ é o termo genérico para obstrução durante a micção. É uma redução do fluxo de urina com aumento simultâneo da pressão detrusora. _NR 3.27_ O índice de obstrução da bexiga (BOOI = P _det_ . Q _max_ - 2Q _max_ ) dará uma orientação para a probabilidade de a obstrução estar presente: ^( 87 )^ BOOI <20cmH _2_ O indica sem obstrução; BOOI 20-40cmH _2_ O indica dúvida e BOOI>40cmH _2_ O indica obstrução. **(** ***Modificado*** **)**
    **3.8.2.2 Micção disfuncional:** caracteriza-se por fluxo intermitente e/ou variável, devido geralmente a inadequado e inconstante relaxamento dos esfíncteres durante a micção em homens neurologicamente normais (ou seja, sem evidência histórica, visível ou mensurável de doença neurológica). **(** ***Modificado*** **)** Micção disfuncional pode causar OIV. Esse tipo de micção também pode ser o resultado de um detrusor acontrátil ou hipoativo (micção com esforço abdominal). A videourodinâmica é necessária para diagnosticar obstrução primária do colo vesical e/ou incoordenação do rabdoesfíncter. ^( 87 )^
    **3.8.2.3 Dissinergia detrusora esfincteriana:** ^( 88 )^ incoordenação entre a função detrusora e do rabdoesfíncter durante a micção, devido a uma alteração neurológica (ou seja, contração do detrusor síncrona com a contração do músculo estriado uretral e/ou periuretral). É uma característica de alterações miccionais de causa neurológica. Alterações neurológicas devem ser pesquisadas. ^( 88 )^ A videourodinâmica **(item 4.3.4)** é geralmente importante para concluir esse diagnóstico. A dissinergia detrusora esfincteriana geralmente ocorre devido a uma lesão acima do nível 3 sacral, mas abaixo da ponte. A eletromiografia do esfíncter pode ser útil se a videourodinâmica não estiver disponível.
    **3.8.2.4** **Obstrução primária do colo da bexiga** **(não neurogênica):** durante a micção, a musculatura lisa do colo vesical falha em abrir adequadamente. O detrusor aumenta a pressão para tentar superar a resistência do colo vesical e permitir que a urina flua ( Figura 7 ).
      **Figura 7:**
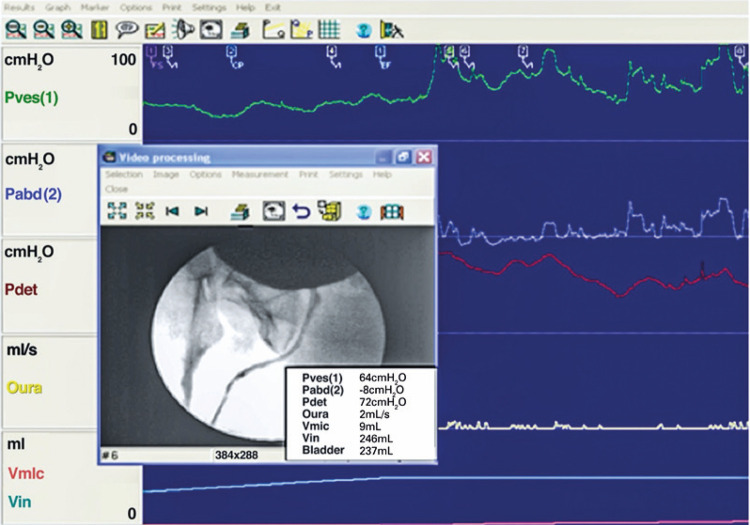
Obstrução primária do colo vesical em paciente não neurogênico: traçado urodinâmico mais imagem

P _ves_: pressão intravesical; P _abd_: pressão abdomina; P _det_: pressão do detrusor.
    **3.8.3 Análise do estudo pressão/fluxo:** apresentação gráfica dos resultados ou cálculos com base na medição da pressão e do fluxo de relação passiva da pressão da uretra foi desenvolvida em nomogramas. Diferentes nomogramas utilizam uma quantidade variável de informação do gráfico de pressão/fluxo. As figuras 8 a 10 [Fig f09] [Fig f10] estão disponíveis para avaliar a OIV nos homens. ^( 89 - 92 )^
      **Figura 8:**
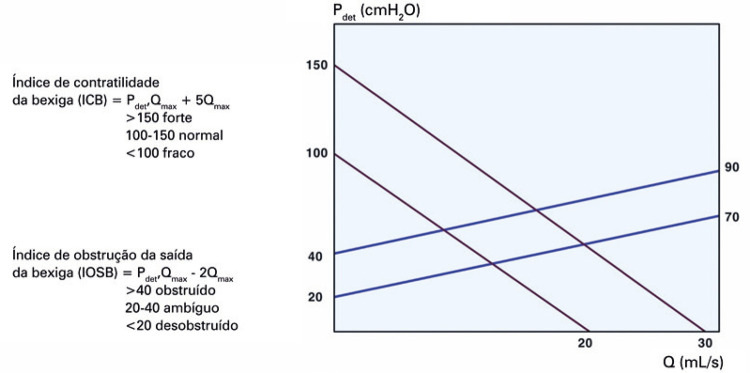
Nomograma da *International Continence Society* (89)

P _det_: pressão do detrusor; Q: fluxo urinário.
      **Figura 9 f09:**
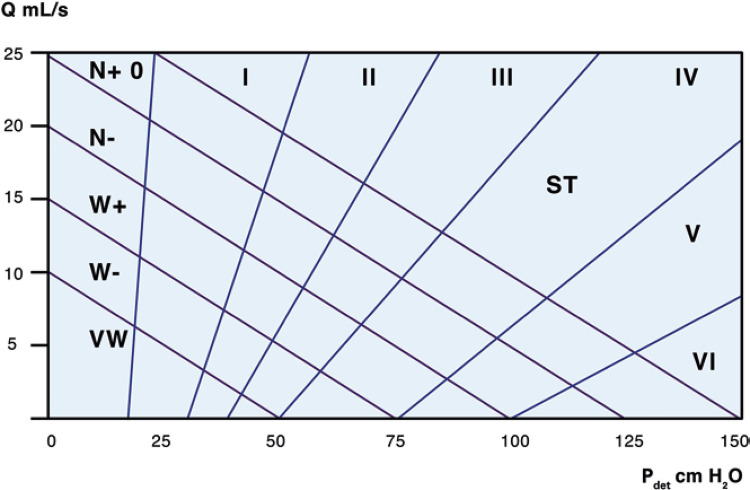
Nomograma de Schäfer (90,91)

P _det_: pressão do detrusor; Q: fluxo urinário.
      **Figura 10 f10:**
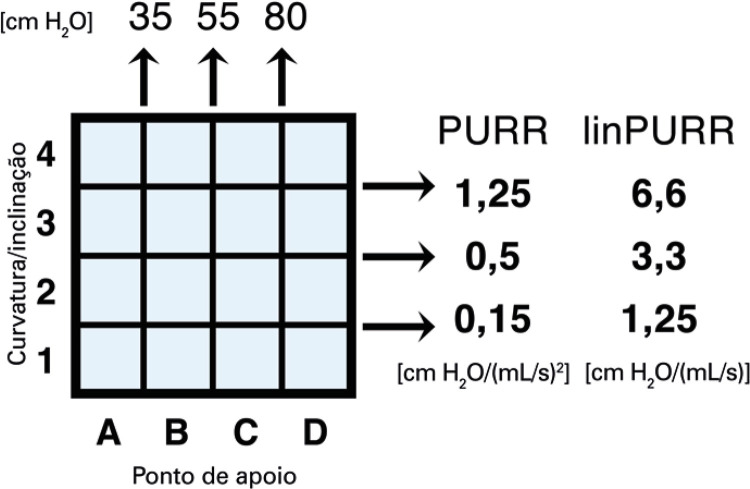
Nomograma de Chess (92) para a classificação bidimensional da obstrução infravesical (avaliação da obstrução infra vesical por compressão e/ou constritiva). Toda a informação do gráfico pressão/fluxo é usada para calcular a relação de resistência uretral passiva (relação de resistência uretral passiva quadrática, isto é, a pressão mais baixa do detrusor para cada fluxo de urina durante o registro da micção; determinação do ponto múltiplo da resistência infravesical). O ponto de referência de relação de resistência uretral passiva (isto é, o ponto de cruzamento da relação de resistência uretral passiva com o eixo de pressão) e a curvatura de relação de resistência uretral passiva (isto é, subida de relação de resistência uretral passiva ) são usados para determinar a resistência infravesical. No total, 16 campos diferentes são gerados usando os valores limites indicados na figura. Apenas o campo A1 testemunha “normal”. Os campos A2 e B1 indicam “dúvida de obstrução”, e todos os outros campos indicam diferentes tipos de obstrução. O aumento dos campos (A a D) indica obstrução infravesical por compressão, enquanto o aumento na curvatura (1 a 4) indica obstrução infravesical constritiva. ( *Novo* )

PURR: *passive urethral resistance relationship* ; linPURR: *linearized passive urethral resistance relationship* .
    **3.8.3.1 Nomograma da ICS:** ^( 89 )^ apenas P _det_ em Q _max_ são representados no nomograma (determinação de um ponto da resistência infravesical). Dependendo da posição desse ponto sobre o nomograma, o paciente pode ser categorizado como “normal”, “equívoco” ou “obstruído”. O cálculo do índice de OIV (BOOI/IOIV) é usado para expressar resistência infravesical como variável contínua. O índice de OIV pode ser extraído do nomograma desenhando uma linha entre a P _det_ , o valor máximo medido da taxa de fluxo urinário e o ponto de corte do eixo Y.
    **NB:** a linha deve ser paralela às traçadas no nomograma, ou seja, aqueles para “normal”, “equívoco” ou “obstruído”. _NR 3.28, NR 3.29, NR 3.30_ **(** ***Novo*** **)**
    **3.8.3.2 Nomograma Schäfer:** ^( 90 , 91 )^ pressão mínima do detrusor na abertura uretral e P _det_ .Q _max_ , juntamente de taxas de fluxo urinário correspondentes, são plotadas no nomograma (determinação de dois pontos de resistência infravesical). A linha entre os dois pontos representa linPURR, e a localização do linPURR no nomograma indica o montante da resistência infravesical do paciente. O nomograma diferencia sete graus de resistência infravesical (graus zero e I: sem OIV; graus II a VI: graus crescentes de OIV). O comprimento (ponto final) de linPURR indica força de contração do detrusor, que pode ser muito fraco (VW), fraco (W), normal (N) ou forte (ST). **(** ***Novo*** **)**
**3.9 Eletromiografia (EMG)**
  **3.9.1 Finalidade:** reflete a atividade da musculatura estriada (periuretral, rabdoesfíncter e AP). O EMG é mal padronizado, devido à variância no tipo de agulha, agulha *versus* eletrodo de contato e local de colocação de eletrodo. ^( 93 )^ Eletrodos de contato perineal são frequentemente preferidos para facilitar a colocação, pela tolerância do paciente e por permitirem maior mobilidade. No entanto, eles medem toda a musculatura estriada da região. Em contraste, os eletrodos de agulha podem ser colocados na área de interesse e medir a atividade de músculos específicos ou grupos musculares, como, por exemplo, o rabdoesfíncter. **(** ***Novo*** **)**
  **3.9.2 Interpretação:** pode ser difícil, devido a artefatos introduzidos por outro equipamento. No contexto urodinâmico, um EMG é útil como indicação grosseira da capacidade do paciente de controlar o AP. **(** ***Novo*** **)**
  **3.9.3 Dissinergia detrusor-esfincteriana (DDE):** contração simultânea do detrusor e esfíncteres uretrais (rabdoesfíncter) com evidência de distúrbio neurológico (seja *deficit* neurológico visível ou mensurável ou uma história de doença neurológica). A classificação de DSD pode ser dividida em dois grupos: contínuo *versus* intermitente. O tipo de dissinergia detrusor-esfincteriana e o grau de lesão medular parecem correlacionar-se. ^( 93 , 94 )^ **(** ***Novo*** **)**
    **3.9.3.1 DDS tipo 1** ocorre em pacientes com lesões neurológicas incompletas. Há aumento progressivo na atividade contrátil do esfíncter urinário externo (rabdoesfíncter), que atinge o pico na contração máxima detrusora, seguida de súbito relaxamento do esfíncter urinário externo, à medida que a P _det_ diminui, permitindo a micção ( Figura 11 ). **(** ***Novo*** **)**
      **Figura 11:**
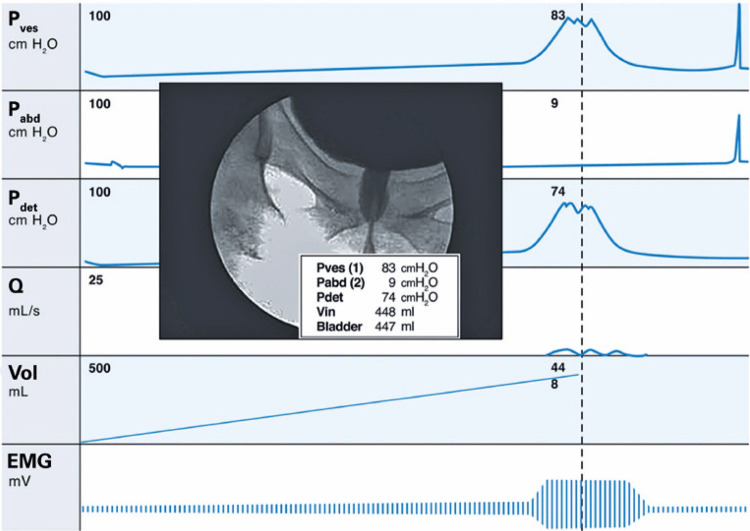
Videourodinâmica com eletromiografia: durante a fase de micção, alta pressão do detrusor, fluxo urinário baixo, aumento da atividade musculatura elétrica e a imagem mostram dilatação da uretra proximal e estreitamento da uretra membranosa (rabdoesfíncter)

P _ves_: pressão intravesical; P _abd_: pressão abdomina; P _det_: pressão do detrusor; Q: fluxo urinário; I _vol_: volume infundido; Vol: volume; EMG: eletromiografia.
    **3.9.3.2 DSD tipo 2** ocorre mais frequentemente em pacientes com lesões completas, com contração contínua do esfíncter urinário externo, ao longo de toda contração detrusora, resultando em obstrução urinária ou incapacidade de urinar. ^( 93 , 94 )^ **(** ***Novo*** **)**
**3.10 Urodinâmica ambulatorial:** teste funcional do TUI, no qual um cateter é colocado na bexiga (e, em alguns protocolos, outro no reto, como é típico no estudo urodinâmico) por via transuretral. É realizada fora do ambiente clínico, envolvendo o enchimento natural da bexiga por ingesta líquida e gravação contínua da P _ves_ por um longo período de tempo (por exemplo: 12 horas). A urodinâmica ambulatorial pode reproduzir a função da bexiga e a perda de urina durante as atividades diárias normais do indivíduo. **(** ***Modificado*** **)**
**3.11 Urodinâmica não invasiva:** o manguito peniano, ^( 95 )^ o cateter de preservativo ^( 96 )^ e o conector uretral ^( 97 )^ foram desenvolvidos como alternativas não invasivas aos estudos de pressão/fluxo. O princípio desses testes é interromper o fluxo e medir a pressão da bexiga.

**Figura 12:**
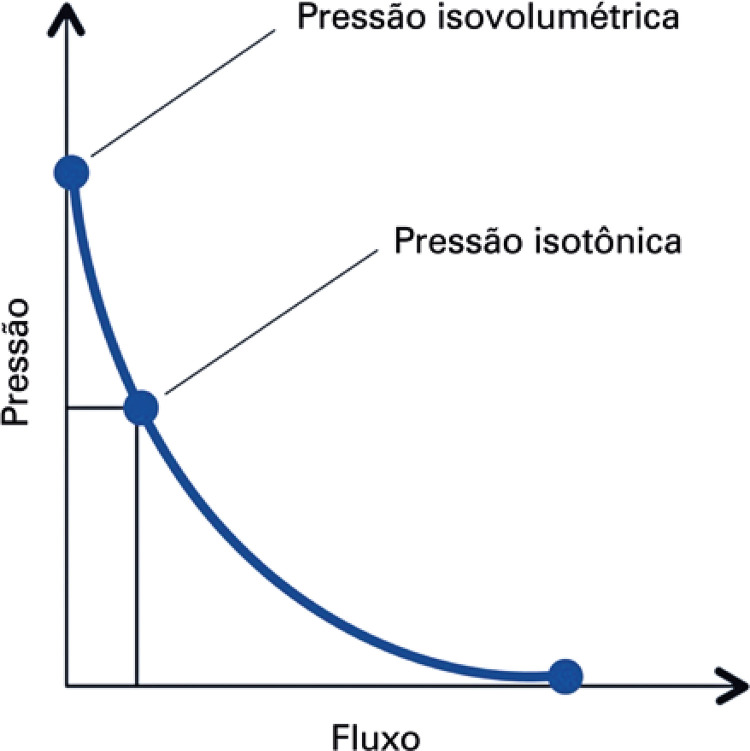
A pressão isométrica e isotônica estão indiretamente relacionadas à condição das fibras musculares (detrusor). A contração isométrica do detrusor, ou seja, a contração sem modificação do comprimento ou sem encurtamento das fibras musculares. A pressão isovolumétrica é estabelecida pela contração isométrica do detrusor (sem fluxo). A contração isotônica desenvolve força com a modificação do comprimento e, portanto, encurtando as fibras musculares. Nesse caso, a pressão isotônica refere-se ao fato de estar sendo desenvolvida na fase de micção

A contração do detrusor é mantida, e os esfíncteres uretrais permanecem abertos. A coluna de líquido da uretra até a bexiga é suficiente para medir a pressão da bexiga (pressão isovolumétrica). A pressão externa sobre a uretra, que é necessária para interromper o fluxo, deve ser idêntica à pressão na bexiga (isto é, pressão isovolumétrica vesical). Portanto, a pressão isovolumétrica vesical fornece informações sobre a P _ves_ durante a micção e, quando o fluxo urinário também é medido, é capaz de distinguir entre obstrução e não obstrução ( Figura 12 ). **( *Novo* )**

**3.12** **Videourodinâmica (fluorourodinâmica):** teste funcional do TUI, no qual a cistometria e o estudo pressão/fluxo (e, possivelmente, a EMG) são combinados com imagens em tempo real do TUI ( Figura 12 ). (ver **item** **4.3.3** ). **(** ***Modificado*** **)**
  **3.12.1 Colo vesical em repouso:** fechado e competente à tosse ou esforço, possível exceção pós-prostatectomia.
  **3.12.2** **Colo vesical durante a micção:** o colo vesical se abre como um funil.
  **3.12.3** **Obstrução do colo vesical durante a micção:** o colo vesical permanece fechado.

## Notas de rodapé para a seção 3

3.1: Urodinâmica é o termo geral para descrever todas as medições que avaliam a função ou disfunção do TUI pela medição de parâmetros fisiológicos relevantes. ^( 56 , 57 )^

3.2: Testes urodinâmicos: ao longo dos anos, uma variedade de termos foram desenvolvidos para o grupo de testes diagnósticos que avaliam a função do TUI: urofluxometria, RPM, cistometria, estudo pressão/fluxo, EMG, perfil de pressão uretral e videourodinâmica (videocisturetrografia) são os termos mais utilizados na literatura científica. ^( 56 , 57 )^

3.3: Homens com hiperatividade detrusora tiveram maiores taxas de fluxo urinário. A hiperatividade do detrusor (anteriormente “instabilidade”) esteve presente em 71% dos homens com classificações de percentis para a taxa de fluxo urinário máximo de 50mL/s. ^( 64 )^

3.4: Existe uma diferença notável entre os nomogramas disponíveis (Liverpool, Siroky e Bristol), particularmente entre raças e em pacientes idosos. ^( 60 - 63 )^

3.5: Estes são valores para a diurese máxima em mulheres em resposta à ingesta de líquido entre 500mL e 1.000mL. Dados equivalentes de diurese masculina não estão disponíveis. ^( 65 )^ Entretanto, a capacidade máxima de diluição da urina é geralmente considerada como 20L/dia, que se converte em 13,9mL/minuto (exatamente o mesmo que os dados femininos. ^( 65 )^

3.6: Nem todos os cateteres esvaziam a bexiga com eficácia semelhante. Há evidências de que, em mulheres, um cateter menos compressível (silicone ou plástico) é muito mais eficaz do que um cateter mais compressível (látex) na drenagem da bexiga. ^( 66 )^ Essa evidência em homens não está disponível.

3.7 Enchimento contínuo de líquido da bexiga por meio de cateter transuretral (ou outra via, por exemplo, cistostomia ou Mitrofanoff), medição da P _abd_ e registro da pressão detrusora, incluindo teste de tosse (esforço). Cistometria termina com “permissão para urinar” ou com incontinência do conteúdo total da bexiga. O tipo de líquido e temperatura, método e taxa de enchimento, tamanhos de cateter, técnica de registro de pressão e posição do paciente devem ser especificados no protocolo urodinâmico.

3.8: Temperatura do líquido infundido, temperatura corporal e temperatura ambiente não afetam diferencialmente os limiares sensoriais da bexiga e nem provocam, de forma desigual, HD ou irritação do TUI. ^( 71 , 72 )^

3.9: A hiperatividade detrusora não teria sido diagnosticada em 76% dos casos de cistometria se tivessem sido feitas na posição supina, e 60% teriam sido perdidos se o estudo fosse feito em posição supina, em relação à sentada. ^( 71 , 73 )^ As posições sentada ou de pé são a mais representativas para situações da vida diária e provavelmente as menos desconfortáveis e/ou embaraçosas para o paciente. ^( 73 )^

3.10: Taxa de enchimento, especialmente quando muito rápida e com volume infundido muito maior que a capacidade funcional vesical, pode influenciar nos resultados ou na representatividade da cistometria. Falta evidência de que a taxa de enchimento deve ser alterada durante a cistometria. Diurese, durante a cistometria, adiciona volume, que não é gravado pelo programa do urodinâmico com a gravação automatizada do volume de enchimento, mas isso é relevante para a interpretação dos resultados.

3.11: Não existe evidência específica, mas, em um estoma, a posição da ponta do cateter é geralmente acima da bexiga, e atividade intestinal pode, com mais frequência, causar artefatos nesses casos, dificultando a mensuração da P _abd_ absoluta e da P _det_ de subtração – e, portanto, a interpretação.

3.12: A pressão urodinâmica é o excesso de pressão acima da atmosfera no nível hidrostático da borda superior da sínfise púbica. Isso é válido para todas as pressões registradas com cateteres preenchidos de líquido.

3.13: Valores avaliados em homens saudáveis ^( 75 , 76 )^ (média ± desvio-padrão) são: primeira sensação de enchimento da bexiga: 222mL±150mL; primeiro desejo de urinar: 325mL±140mL e forte desejo de urinar: 453mL±94mL.

3.14: Capacidade cistométrica máxima que, em homens adultos saudáveis, deve ser, em média, de 552mL (faixa 317mL a 927mL). ^( 76 )^

3.15: O enchimento com de mais de 800mL raramente é útil. ^( 71 )^

3.16: Capacidade vesical máxima sob anestesia (“capacidade anatômica da bexiga”). O volume ao qual a bexiga pode ser preenchida em anestesia geral profunda ou espinhal, sem perda urinária, raramente é relatado na literatura científica, mas pode ser de relevância na cistite intersticial. ^( 71 )^

3.17: “Detrusor normoativo” como vários estudos demonstraram HD durante o enchimento em indivíduos saudáveis.

3.18: A ITU é uma causa muito incomum de HD. A maioria dos centros não realiza estudo urodinâmico na presença de infecção ativa por causa do risco de septicemia.

3.19: Valores normais de complacência vesical em homens não foram bem definidos. Complacência vesical em voluntários foi maior do que geralmente considerado normal em adultos durante a cistometria. ^( 76 )^ Em 28 voluntários saudáveis do sexo masculino, com idade média de 24 anos (variação de 19 a 28), a complacência média foi de 56,1mL/cm H _2_ O (desvio padrão de 37,3). Visto que não existem valores precisos para a complacência normal nos homens, um estudo prospectivo de uma grande população normal é necessário.

3.20: Não há evidências convincentes de que o diagnóstico clínico com base na primeira cistometria é frequentemente alterado na repetição do teste. Não há evidência definitiva de que a repetição imediata de um teste urodinâmico “para confirmação” seja necessária. A recomendação de repetição imediata do teste é para quando existe dúvida sobre se o teste respondeu à questão clínica e se erros técnicos e artefatos forem observados na análise imediata pós-teste.

3.21: Cistometria e estudo pressão/fluxo, urofluxometria livre e RPM são denominados padrões urodinâmicos da ICS (ICS-SUT). Isso pode ser complementado com outros testes, como EMG, imagenologia, pressão uretral contínua e/ou medidas do perfil de pressão uretral. Todos os testes são realizados na posição preferida dos pacientes ou na posição mais usual: confortavelmente sentado e/ou de pé, se possível. ^( 56 , 57 )^

3.22: A fisiologia da micção depende da ativação neural central, da contratilidade vesical e do relaxamento uretral coordenado durante todo o processo. Ainda resta muito a aprender sobre esses componentes, incluindo ativação central, sua classificação potencial e seu papel e interações na HAD e na micção disfuncional.

3.23: Geralmente é entre 0,5 a 0,8 segundo, dependendo da posição do indivíduo e da distância até o urofluxômetro.

3.24: O primeiro “evento” na micção é o relaxamento do AP. Isso pode significar queda na pressão intra-abdominal e aumento associado da P _det_ , que não implica em uma contração detrusora.

3.25: Como qualquer outra contração muscular, a contração detrusora tem componente isométrico e outro isotônico. O componente isométrico significa que as fibras detrusoras não encurtam e a P _ves_ aumenta. O componente isotônico produz mudanças no comprimento da fibra; há, sim, encurtamento e um fluxo segue. O primeiro é representado externamente como P _ves_ ou P _det_ e o segundo, por fluxo. Na fase miccional, na presença de fluxo, a pressão detrusora é uma função dessas duas variáveis, governadas pela resistência uretral ao fluxo.

3.26: Interrupção voluntária da micção: se surgir a necessidade de interromper o fluxo, a contração do AP e dos esfíncteres uretrais pode fazer isso, resultando em aumento isométrico da P _det_ . Urina na uretra proximal é ordenhada de volta para a bexiga.

3.27: Em homens com sintomas de disfunção do TUI, fluxo urinário (taxa) e RPM são importantes marcadores de OIV, mas também são dependentes da central de iniciação e continuação da contração e pressão detrusoras. Na definição original, apenas pressão e fluxo de urina foram incluídos.

3.28: Apresentação gráfica da micção: foi recomendado apresentar estudos de pressão/fluxo com gráfico da taxa de fluxo (mL/s) no eixo X e pressão síncrona do detrusor (cm H _2_ O) no eixo Y, além dos gráficos com base no tempo, mas os eixos podem ser invertidos. A esses gráficos pode ser adicionado um valor de corte ou faixa de normalidade, além de zonas equívocas. Esses valores de corte são específicos da população, variando amplamente entre os pacientes do sexo masculino.

A relação entre pressão detrusora e fluxo síncrono gerado indica “resistência uretral”. Com auxílio da computação, esses gráficos podem ser desenhados desde o início até o final do fluxo. A resistência uretral é, então, apreciada graficamente, ao longo de toda a fase de esvaziamento. A maioria desses pontos de resistência são considerados impulsionados por atividade muscular uretral. O ponto de menor resistência calculada deve ser tomado como uma aproximação à resistência uretral livre da atividade muscular uretral e das contrações periuretrais. Esse conceito de “relação de resistência uretral passiva” é tomado como “obstrução anatômica” causada por estruturas fixas, como a próstata ou estenoses.

Gráficos de pressão/fluxo como medida de contração detrusor durante a micção. “Contratilidade detrusora” pode ser usada para qualquer método que diagnostica ou pretende diagnosticar propriedades “intrínsecas” do músculo detrusor (por exemplo, força ou velocidade potencial máxima), por qualquer método. Em um determinado grupo de pacientes, a contratilidade do detrusor pode ser calculada em séries de interrupção do fluxo ou testes de interrupção da micção e análise matemática ou, ainda, gráfica dos métodos de pressão, fluxo e outros parâmetros. Valores de corte ou uma escala contínua de contratilidade podem ser desenhados. Independentemente da magnitude da contração detrusora, esta pode estar desaparecendo antes do esvaziamento total, levando à micção incompleta; “contração não sustentada” ou “contração enfraquecida” podem, então, ser usadas.

3.29: Nomograma ICS ^®^ , anteriormente conhecido como nomograma de Abrams-Griffiths e número de Abrams-Griffiths (agora BOOI), é mais comumente usado.

3.30: O fluxo com cateter na uretra deve ser comparado com fluxo livre, para verificar se a micção disfuncional pode ter ocorrido somente durante a urodinâmica, devido ao cateter.

## SEÇÃO 4 – IMAGEM

**4.1 Visão geral:** Os exames de imagem tornaram-se cada vez mais importantes na avaliação do TUI e na disfunção do AP masculino. A tabela 2 indica as possíveis modalidades de exames por localização e os principais objetivos dos rins ao AP. **(** ***Novo*** **)**

A aplicação da técnica de imagem específica é dependente da suspeita de anormalidade, da capacidade da técnica de imagem na visualização dessa anormalidade e da resolução da imagem. No caso de avaliações que competem em sua indicação, técnicas não radiológicas devem ser priorizadas para evitar exposição à radiação. **( *Novo* )**

**4.2 Ultrassonografia**
  **4.2.1** **Ultrassonografia na avaliação do trato urinário inferior**

Como observado na tabela 2 , a ultrassonografia tornou-se uma modalidade relevante em todos os órgãos que possam estar sujeitos à investigação do TUI masculino e da disfunção do AP, tanto no consultório como associada ao estudo urodinâmico. **( *Novo* )**

**Tabela 2:** Modalidades de exame de imagem e objetivos

Localização	Técnica de imagem	Objetivo
Trato urinário superior	Ultrassonografia renal Urografia excretora, urotomografia ou urorressonância	Detectar a presença e o grau de hidronefrose, carcinomas/tumores uroteliais, cálculos, outras anormalidades renais e ureterais
Bexiga	Ultrassonografia da bexiga Ultrassonografia transabdominal Ultrassonografia transretal TC RM	Medida do resíduo pós-miccional, espessamento da parede vesical e detrusor, protrusão intravesical da próstata ou peso vesical (para auxiliar no diagnóstico de obstrução infravesical) ou calcificações Avaliar a presença de outras alterações como neoplasias, cálculos ou corpo estranho
Próstata	Ultrassonografia transretal (tridimensional, otimizada com contraste e Doppler) Ultrassonografia transabdominal Ressonância magnética (ponderada em T2, multiparamétrica, segmentação prostática e ressonância funcional)	Avaliar volume prostático Anatomia da próstata Obstrução infravesical RM: avaliação de câncer de próstata
Escroto	Ultrassonografia escrotal	Testículos, epidídimo e túnica vaginal
Uretra	Raios X (uretrocistografia retrógrada ou miccional) RM Ultrassonografia	Avaliar anormalidades congênitas, fístulas, divertículo, estenoses (pós-operatória) e neoplasias
Ânus e reto	Ultrassonografia endoanal (10, 13 e 16Hz de campo axial ou sagital)	Integridade do esfíncter anal, abcesso perianal e coordenação do assoalho pélvico durante a defecação
Pênis	Ultrassonografia TC RM	Doença de Peyronie, ruptura dos corpos cavernosos
Trato urinário inferior	Videouretrocistografia Videourodinâmica	Avaliar a bexiga durante o enchimento e/ou esvaziamento, refluxo vesicoureteral, morfologia vesical (trabeculações e divertículos), localização da obstrução infravesical, tipo de incontinência de esforço, divertículo de uretra, estenoses e fístulas

TC: tomografia computadorizada; RM: ressonância magnética.

**4.2.2** Modalidades atuais no uso em rotinas clínicas:
  **4.2.2.1 Transretal:** com transdutor linear ou endoretal. **(** ***Novo*** **)**
  **4.2.2.2 Transabdominal:** transdutor curvo ou linear aplicado no abdômen. **(** ***Novo*** **)**
  **4.2.2.3 Perineal**: transdutor curvo ou linear aplicado ao períneo (transperineal). **(** ***Novo*** **)**
  **4.2.2.4 Escrotal:** transdutor linear aplicado no escroto para avaliar testículos, epidídimos e anormalidades intraescrotais. **(** ***Novo*** **)**
**4.2.3** Indicações atuais da ultrassonografia na avaliação de STUI e disfunção do AP em homens
  **4.2.3.1 Resíduo pós-miccional (RPM):** medição ultrassonográfica do volume da bexiga via transabdominal ^( 98 , 99 )^ ou transretal ^( 100 )^ **(item 3.2.2).** A seguinte fórmula mostra menor taxa de erro pela via transabdominal quando comparada com a cateterização: ^( 99 )^ O cálculo do RPM (por ultrassonografia abdominal) é feito multiplicando a largura (entre as bordas da esquerda para a direita), profundidade (das bordas anterior a posterior) e comprimento (limites cranial a caudal) e multiplicando esse resultado por 0,52 (há diferentes fatores de multiplicação disponíveis, mas 0,52 é o mais comum) ( Figura 13 ). **(** ***Novo*** **)** volume = (largura x profundidade x comprimento [cm]) x 0,52mL
    **Figura 13:**
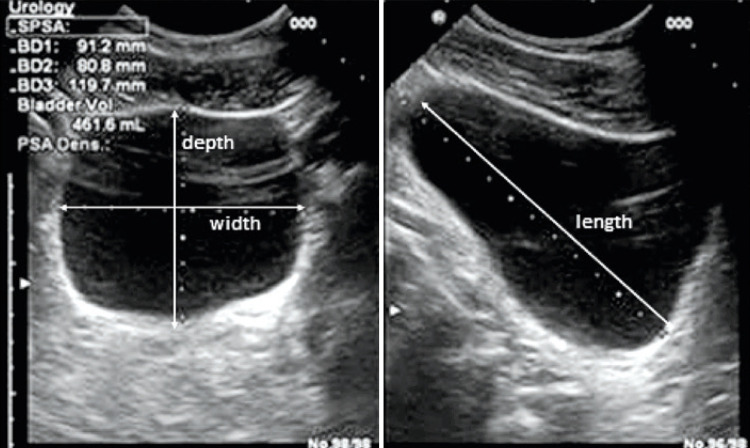
Determinação do volume da bexiga (residual pós-miccional) por ultrassom abdominal
  **4.2.3.2 Anormalidades acidentais:** por exemplo: volume da próstata (transabdominal, tumor intra-abdominal ou tumor retroperitoneal e hidronefrose). **(** ***Novo*** **)**
  **4.2.3.3 An** **omalias da bexiga**: por exemplo, tumor, corpo estranho, hiperdistensão e cálculos vesicais. **(** ***Novo*** **)**
  **4.2.3.4 Espessura da parede do detrusor ou espessura da parede da bexiga:** Visualização por via abdominal (suprapúbica) da parede anterior da bexiga com transdutor linear de alta frequência para a detecção de OIV: se espessura da parede do detrusor ≥2mm em bexigas com repleção de ≥250mL ( Figura 14 ) ou espessura da parede da bexiga ≥5mm em bexigas com repleção de 150mL ( Figura 14 ). ^( 101 - 105 )^ **(** ***Novo*** **)**
    **Figura 14:**
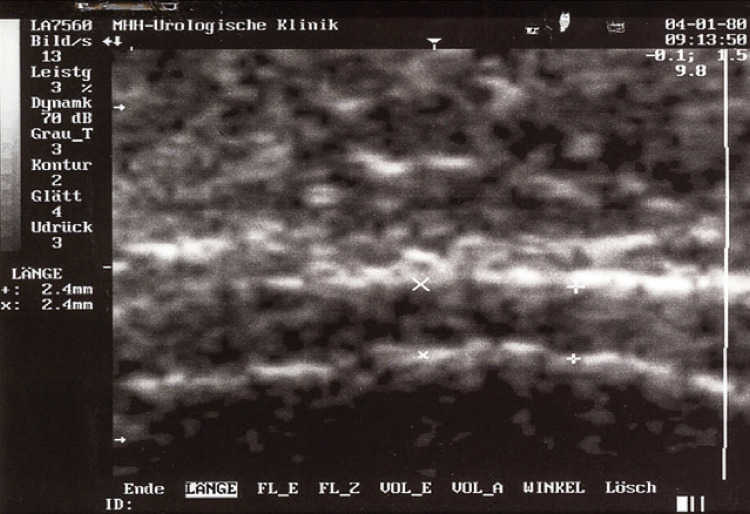
Medição por ultrassom da espessura do detrusor na parede anterior da bexiga com ultrassom linear de 7,5MHz em uma bexiga cheia >250mL. O detrusor hipoecogênico (barra escura) é imprensado entre a mucosa hiperecogênica (branca) (parte inferior) e a adventícia (parte superior). (101,102) A espessura do detrusor é medida da borda interna da mucosa até a borda interna da adventícia, como demonstrado na figura, enquanto a espessura da bexiga é medida da borda externa da mucosa até a borda externa da adventícia
  **4.2.3.5 Peso vesical estimado por ultrassom:** pode ser calculado medindo o volume de urina na bexiga e a espessura da parede vesical, aplicando-se a fórmula apresentada na figura 15 . ^( 106 , 107 )^ **(** ***Nov*** ***o*** **)**
    **Figura 15:**
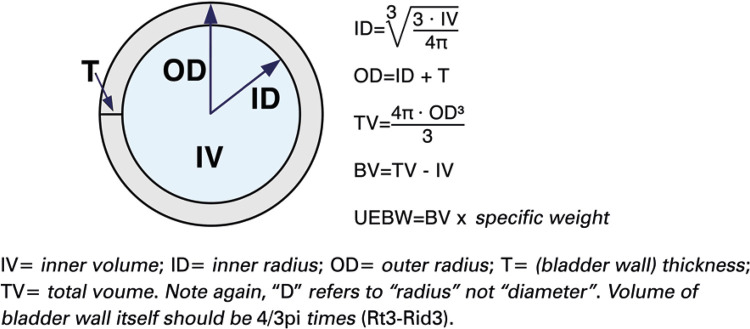
Peso vesical estimado por ultrassom. (106,017) NR 4.1 Observe novamente que “D” refere-se a “raio”, e não ao “diâmetro”. O volume da parede da bexiga em si deve ser 4/3pi vezes (Rt3-Rid3)
  **4.2.3.6 Protrusão prostática intravesical (IPP):** medição transabdominal da distância da base da bexiga até a borda da próstata na luz da bexiga ^( 108 )^ ( Figuras 16A e 16B ). É recomendado encher a bexiga com 100mL a 200mL de líquido para oferecer medições representativas. Enchimento vesical maior que 400mL diminui os valores da IPP. ^( 108 )^ A medição da IPP pode ser dividida em três graus: grau I, de zero a 4,9mm; grau II, de 5mm a 10mm; grau III, >10mm. ^( 109 )^ A IPP grau III é associada com IOV relacionada à próstata.
    **Figura 16:**
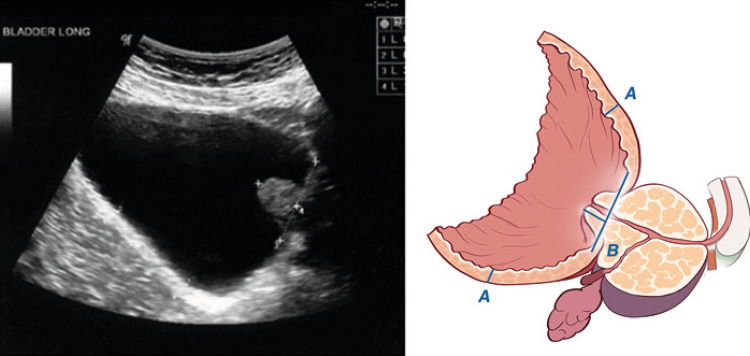
A) Medição pelo ultrassom transabdominal da protusão prostática intravesical (IPP). B) Como medir IPP - base da bexiga (linha A) para a parte mais cranial da próstata (linha B)
  **4.2.3.7 Anormalidade uretral:** por exemplo: divertículo, estenose uretral, desnivelamentos, e profundidade da espongiofibrose. **(** ***Novo*** **)**
  **4.2.3.8** **Resultados pós-operatórios:** por exemplo: pós-prostatectomia (formato uretral), posição do *sling* masculino, posicionamento do manguito e reservatório do esfíncter urinário artificial e agentes preenchedores. **(** ***Novo*** **)**
  **4.2.3.9 Ultrassonografia da próstata:** determinação do volume e da zona de transição prostática, forma da próstata e visualização do parênquima da próstata para calcificações, cistos, abscessos ou aumento de volume ( Figura 17 ). **(** ***Novo*** **)**
    **Figura 17:**
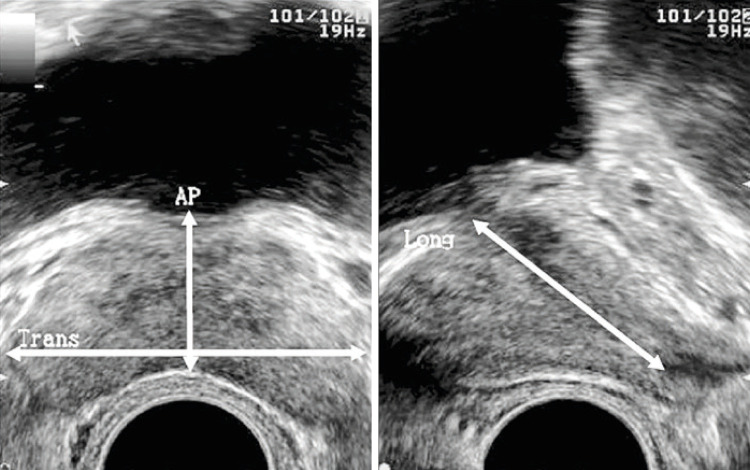
Volume prostático por ultrassonografia transretal
**4.2.4 AP: por exemplo:** defeitos do esfíncter anal
**4.2.5** Ultrassonografia em três e quatro dimensões: modalidades de pesquisa presente. _NR 4.2_
**4.2.6 Outras avaliações:** Ultrassonografia sincrônica da bexiga/uretra associada à aferição da P _abd_ e vesical, durante o enchimento e no estudo de fluxo/pressão (vídeo-ultrassom-urodinâmica). **(** ***Novo*** **)**
**4.2.7 Ultrassonografia anal (endossonografia):** ^( 110 , 111 )^ Esta é a investigação padrão-ouro na avaliação da integridade do esfíncter anal. É alta a incidência de sintomas defecatórios em homens com defeitos do esfíncter anal ( Figura 18 ). **(** ***Novo*** **)**
  **4.2.7.1 Ultrassonografia endoanal ou endossonografia anal:** Ultrassom do canal anal é realizado com uma sonda de ultrassom tipo transretal colocada no canal anal, produzindo imagem em 360 ^o^ do canal anal. O exame é geralmente realizado com o paciente colocado na posição de litotomia, prona ou decúbito lateral esquerdo. Endossonografia bidimensional e endossonografia tridimensional (reconstrução tridimensional do canal anal) são realizadas usando tanto imagens axiais quanto sagitais. **(** ***Novo*** **)**
  **4.2.7.2 Canal anal:** o canal anal em adultos tem entre 2,5cm e 5cm de comprimento e começa quando o reto se estreita, passando posteriormente entre os elevadores do ânus. Há três níveis de avaliação no plano axial. ^( 111 )^ **(** ***Novo*** **)**
  **4.2.7.2.1 Nível superior:** formado pela alça hiperecoica do músculo puborretal e pelo anel completo do esfíncter anal interno (EAI). **(** ***Novo*** **)**
  **4.2.7.2.2 Nível médio:** corresponde à parte superficial do esfíncter anal externo (EAE) (banda concêntrica de ecogenicidade mista), a camada longitudinal conjunta, o EAI (anel concêntrico hipoecóico) e músculos transversais do períneo. **(** ***Novo*** **)**
  **4.2.7.2.3 Nível inferior:** corresponde à parte subcutânea do EAE, na qual o EAI está ausente. **(** ***Novo*** **)**
  **4.2.7.3 EAI:** a continuação caudal do músculo circular do reto forma o EAI, que termina caudalmente em um limite claramente definido, numa distância variável da borda anal. ( ***Novo*** **)**
  **4.2.7.4 Músculo longitudinal:** compreende células musculares lisas contínuas com a camada externa da parede retal e músculo estriado de vários músculos do AP. O músculo longitudinal encontra-se entre os esfíncteres anais interno e externo no espaço interesfinctérico. **(** ***Novo*** **)**
  **4.2.7.5 Esfíncter anal externo:** é composto de músculo estriado e envolve o músculo longitudinal, formando a borda exterior do espaço interesfincteriano.O esfíncter externo é dividido em três partes (profunda, superficial e subcutânea), com as porções profunda e subcutânea formando anéis musculares, entre as fibras elípticas da parte superficial do EAE se direcionando anteriormente do corpo perineal à região do cóccix posteriormente. **(** ***Novo*** **)**
  **4.2.7.6 Puborretal:** é formado a partir de fibras anteriores do músculo pubococcígeo, perfazendo uma alça que puxa o reto para região anterior. **(** ***Novo*** **)**
  **Figura 18:**
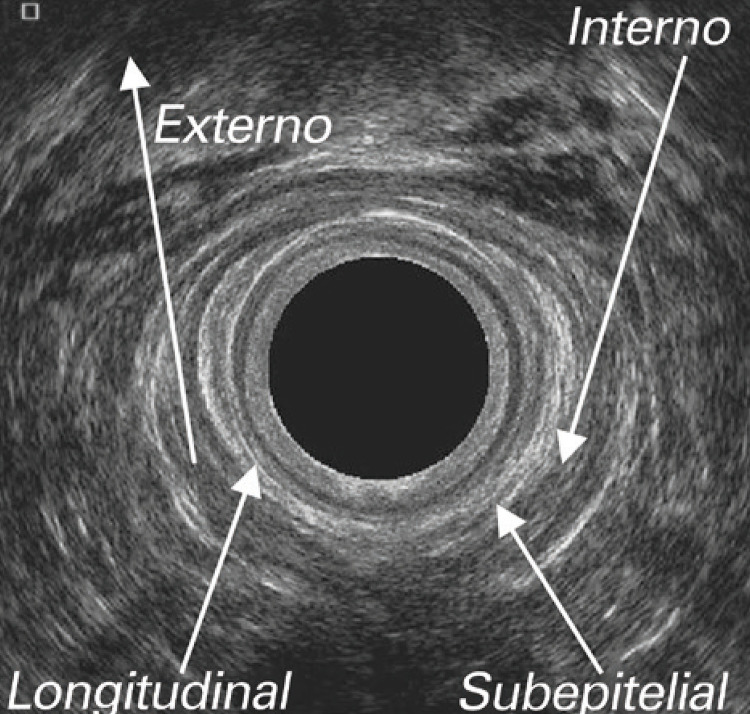
Anatomia normal do canal anal como vista na endossonografia anal
**4.3 Radiografia**
  **4.3.1 Modalidades atuais em uso clínico rotineiro**
    **4.3.1.1 Urografia intravenosa:** fornece descrição anatômica do trato urinário superior, dos ureteres e da bexiga, bem como a avaliação da função renal e da excreção, do meio de contraste. Urografia intravenosa consiste em pelo menos três a quatro imagens abdominais: um raios X simples, um quase imediatamente após a injeção de avaliar para captação vascular renal, uma imagem aos 7 minutos e outra aos 15 minutos após a infusão de meio de contraste (e esvaziamento de bexiga). A radiografia simples preliminar pode mostrar calcificação no rim, ureter, bexiga, vesículas seminais ou vasos. **(** ***Novo*** **)**
    **4.3.1.2 Uretrocistografia retrógrada e uretrocistografia miccional:** exame de imagem com contraste unidirecional ou combinado da uretra com paciente em posição oblíqua de 30° para visualizar a luz uretral, principalmente para diagnosticar estenoses uretrais ou divertículos ( Figura 19 ). Também é útil para diagnosticar e estadiar trauma uretral. **(** ***Novo*** **)**
      **Figura 19:**
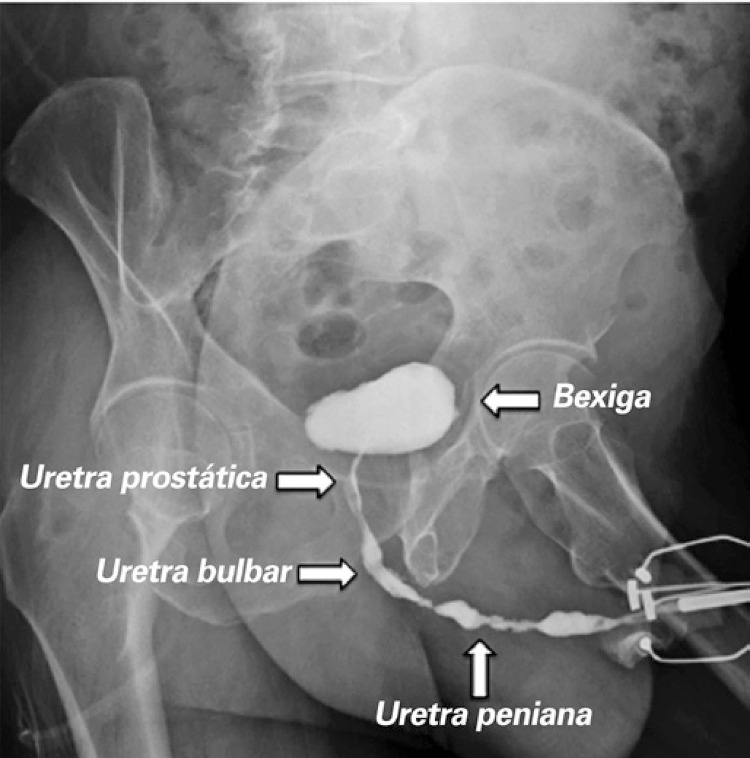
Uretrocistografia retrógrada de paciente com estenose da uretra peniana
    **4.3.1.3 Uretrocistografia miccional:** estudo de imagem do colo da bexiga, uretra e próstata durante a micção ( Figura 20 ). O principal uso é a determinação da localização de qualquer obstrução como, por exemplo, o colo da bexiga ou a próstata. Este exame pode detectar o refluxo vesicopieloureteral, fístula vesical ou uretral, divertículos uretrais e/ou vesicais e estenoses. **(** ***Novo*** **)**
      **4.3.1.3.1 Videocistouretrogra** **fia**: trata-se de uma uretrocistografia com captura de imagem contínua ( Figura 21 ). **(** ***Novo*** **)**
        **Figura 21:**
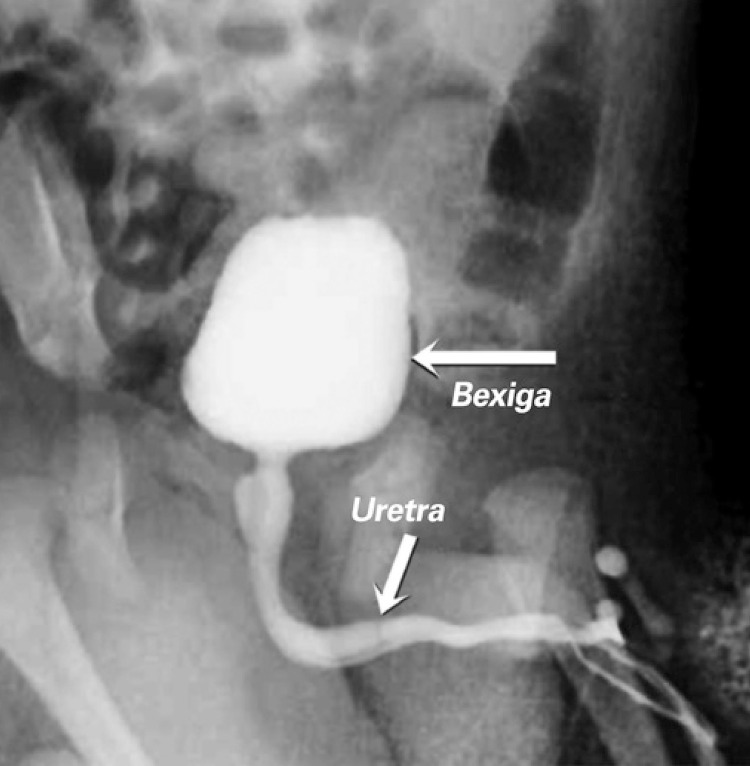
Exemplo de videocistouretrografia
      **Figura 20:**
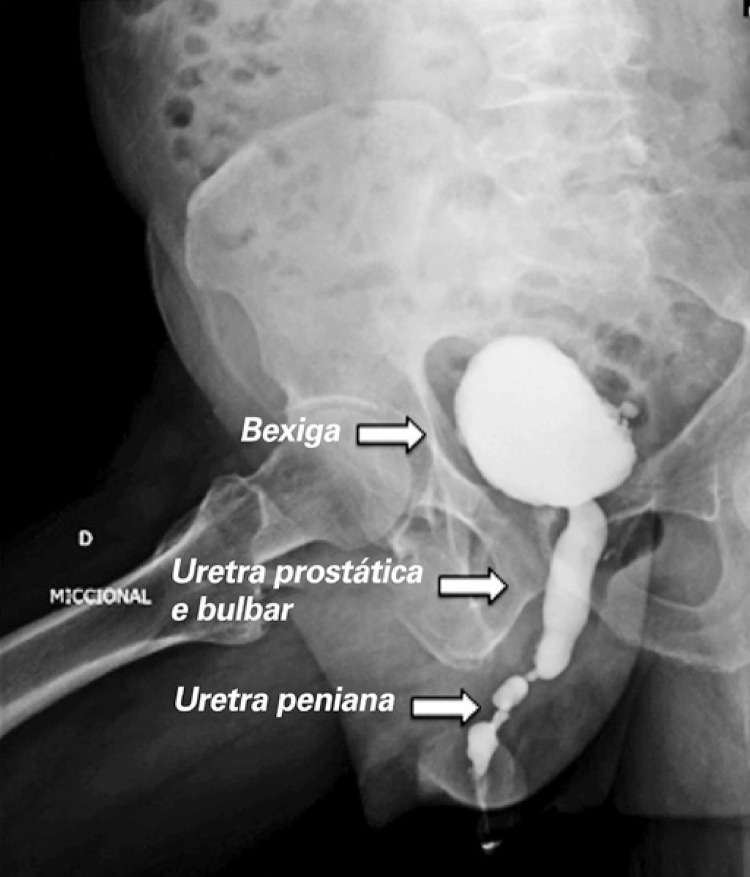
Cistouretrografia miccional: mostra divertículos da bexiga, colo da bexiga aberto e uretra prostática dilatada até estenose da uretra peniana
  **4.3.4 Videou** **rodinâmica:** ^( 112 )^ videourodinâmica refere-se à videouretrocistografia com registro sincrônico da pressão e da taxa de fluxo miccional. É um estudo dinâmico com a função de estudar a bexiga durante seu enchimento e esvaziamento ( Figura 12 ). **(** ***Novo*** **)** A videourodinâmica tem duas características definidas: - É uma técnica cinética que registra alterações morfológicas e funcionais do TUI em função do tempo. Esse recurso distingue essa técnica das imagens estáticas obtidas por cistografia. - É uma técnica que é aplicada simultaneamente com o estudo urodinâmico convencional. A aquisição de imagem para o trato urinário pode ser realizada com raios X (fluoroscopia) ou ultrassom – embora, em sentido estrito, o prefixo “vídeo” refere-se à gravação das imagens, e não à sua aquisição.
  **4.3.5** **Defecografia (Proctografia evacuatória):** Este exame demonstra a anatomia anorretal, bem como distúrbios da evacuação retal. Contraste de Bário é inserido no reto antes de defecação sobre um vaso sanitário translúcido. **(** ***Novo*** **)**
**4.4 Tomograf** **ia computadorizada**
  **4.4.1 Urografia computadorizada (TC urografia):** estudo de TC do sistema urinário utilizando injeção de contraste para esclarecer diagnósticos, como (i) tumores, (ii) doença renal, coleções líquidas anormais/abscessos e (iii) doenças da bexiga. **(** ***Novo*** **)**
  **4.4.2 Urotomografia (TC sem contraste):** estudo de imagem por tomografia sem contraste principalmente indicado para identificar cálculos, mas pode identificar outras doenças. Também conhecido como “protocolo para litíase”. **(** ***Novo*** **)**
**4.5 Estudo de imagem por RM** ^( 113 )^
  **4.5.1 RM na disfunção do TUI e do AP masculino:** A RM fornece a oportunidade de examinar as estruturas de tecidos moles da pelve e estruturas de suporte. É não invasiva, tem excelente resolução de contraste em tecidos moles, sem exposição à radiação ionizante e permite o estudo da função das estruturas do AP sob diferentes condições dinâmicas. Vários pontos anatômicos usados para medições pélvicas também são facilmente identificados na RM e a maioria das medições são, portanto, altamente reprodutíveis. Ponderação em T auxilia no realce das estruturas, conforme densidade hídrica. **(** ***Novo*** **)**
  **4.5.2** Atuais aferições possíveis no uso da RM no TUI masculino e na disfunção do AP. _NR 4.2_
    **4.5.2.1 Anomalias da bexiga:** por exemplo: tumor, corpo estranho, anormalidades da parede vesical e fístula enterovesical. **(** ***Novo*** **)**
    **4.5.2.2 Anormalidade uretral:** por exemplo: divertículo e fístula retouretral. **(** ***Novo*** **)**
    **4.5.2.3 Tamanho do esfíncter uretral:** ^( 114 )^ predição de incontinência urinária pós-prostatectomia. **(** ***Novo*** **)**
    **4.5.2.4 Anormalidades da próstata:** por exemplo: hiperplasia benigna, câncer, cistos e fístula prostatorretal. **(** ***Novo*** **)**
    **4.5.2.5 Anomalias intercorrentes:** por exemplo: reto (a dinâmica retal pode ser avaliada durante evacuação, após a adição de gel de ultrassom no reto). Os movimentos do AP e anorretal podem ser avaliados oferecendo imagens em repouso e no esforço. **(** ***N*** ***ovo*** **)**
    **4.5.2.6 Anormalidades congênitas:** detecção de ductos müllerianos remanescentes, aberrações na inserção dos ureteres e duplicação de estruturas pélvicas. **(** ***Novo*** **)**
    **4.5.2.7 Padronização do estudo de imagem da próstata por RM:**
    Próstata *Prostate imaging reporting and Data System* (PI-RADS) e sistema de dados ( Figuras 22-24 [Fig f23] [Fig f24] ). _NR 4.4, NR 4.5_ **(** ***Novo*** **)**
      **Figura 22:**
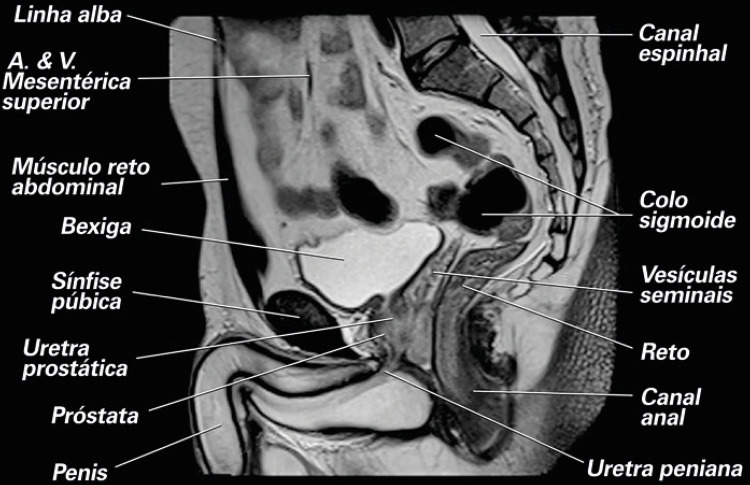
Ressonância magnética (sagital) do abdômen inferior e pelve
      **Figura 23 f23:**
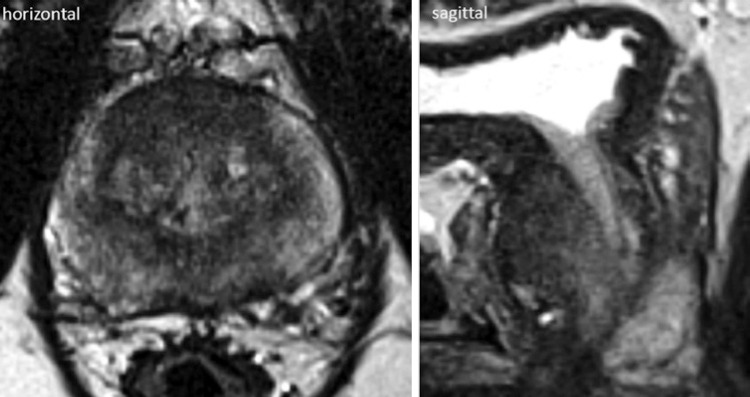
Ressonância magnética da próstata mostrando alterações inflamatórias de baixo grau na zona periférica
      **Figura 24 f24:**
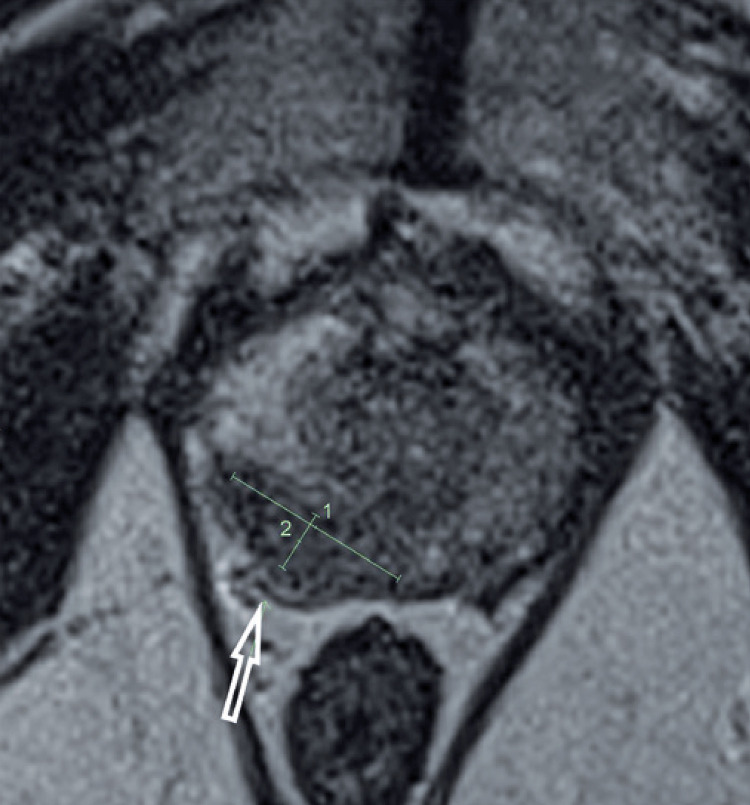
Ressonância magnética da próstata mostrando câncer de próstata na zona periférica posterolateral direita

## Notas de rodapé para a seção 4

4.1: O valor de “corte” para obstrução tem sido sugerido como 35g (homens adultos asiáticos). ^( 106 )^

4.2: O potencial do ultrassom em três e quatro dimensões no estudo do trato urinário baixo e disfunção do AP em homens vem sendo pesquisado atualmente com aplicações validadas e pode ser incluído em atualizações futuras deste relatório e/ou em relatórios em separado.

4.3: Capacidade diagnóstica pode ser melhorada utilizando-se RM tridimensional. Novas técnicas com sequência de alta velocidade de fotos permitem uma ressonância funcional.

4.4: Estudos de imagem da próstata tornaram-se mais padronizados nos últimos 5 anos com a introdução do PI-RADS, atualmente em sua versão 2.

O protocolo recomendado de RM de próstata consiste em estudo multiparamétrico com pelo menos uma sequência de difusão (DWI), sequências de alta resolução anatômica ponderadas em T2 e sequências avançadas com imagens de perfusão.

Pontuação de PI-RADS finalmente é dada com base em um esquema de registro estruturado. Uma pontuação de um a cinco é dada, sendo um para benigno e cinco para altamente suspeito de malignidade.

Idealmente, os estudos de RM são feitos em um equipamento de Tesla 3 de força sem a necessidade de uma bobina endorretal para obter uma resolução adequada.

Atualmente, a espectroscopia por imagem de RM na próstata é raramente executada e pouco acrescenta valor ao estudo multiparamétrico.

## SEÇÃO 5 – DIAGNÓSTICOS (MAIS COMUNS)

Este relatório, como os anteriores, ^( 2 , 3 , 5 )^ destaca a necessidade de ter como base os diagnósticos para disfunção do TUI e do AP masculino na correlação entre sintomas, sinais e quaisquer investigações diagnósticas relevantes, foram incluídas EMG e imagens como possíveis investigações diagnósticas. Os diagnósticos são categorizados de acordo com três subgrupos que refletem a função do TUI, ou seja, disfunção de armazenamento e esvaziamento e disfunção mista de armazenamento e esvaziamento. São escassos os dados de prevalência para a frequência relativa dos diferentes diagnósticos masculinos. _NR 5.1_ Mais estudos são necessários. **( *Novo* )**

**DISFUNÇÃO DE ARMAZENAMENTO**_NR 5.1_ Esses diagnósticos relacionados a alterações anormais na sensação da bexiga, P _det_ ou capacidade da bexiga durante a cistometria da fase de enchimento.

**5.1 Fator vesical**
  **5.1.1 Hipersensibilidade vesical** ^( 5 )^_NR 5.2, NR 5.3_ **(** ***Novo – masculino*** **)**
    **5.1.1.1 Definição:** a hipersensibilidade vesical é um diagnóstico clínico feito por ***sintomas e investigações urodinâmicas,*** com maior probabilidade de ocorrer em indivíduos com sintomas de aumento da frequência diurna e noctúria. Uma tabela de volume e frequência mostra VU médio reduzido (dia e noite). Como observado no **item 3.4.3.6,** ele pode ser definido como aumento da sensação de bexiga percebida durante o enchimento da bexiga com achados cistométricos específicos de desejo precoce de esvaziar **(3.4.3.2);** forte desejo precoce de esvaziar, que ocorre em baixo volume vesical **(3.4.3.4);** baixa capacidade cistométrica máxima vesical **(item 3.4.4.2)** e nenhum aumento anormal na P _det_ . Volumes vesicais específicos nos quais esses achados ocorrem variam em diferentes populações. _NR 5.3_
  **5.1.2 Hiperatividade detrusora:**_NR 5.1, NR 5.4, NR 5.5, NR 5.6_
    **5.1.2.1 Definição**: conforme observado no **item 3.4.5.2,** esse diagnóstico por ***sintomas e investigações urodinâmicas*** é feito em indivíduos com sintomas do TUI (mais comumente sintomas da BH **(item 1.1.7),** quando ocorrem contrações do músculo detrusor durante a cistometria. **(** ***Modificado*** **)**
    **5.1.2.2** Subtipos
    **(i)** **Hiperatividade detrusora idiopática (primária):** como observado no **item 3.4.5.2.1,** não há causa identificável para as contrações involuntárias do detrusor. **(** ***Modificado*** **)**
    **(ii)** **Hiperatividade detrusora neurogênica (secundária):** ^( 3 , 5 , 13 )^ como observado no **item 3.4.5.2.2,** há HD e evidência (história e *deficit* visível ou mensurável) de um distúrbio neurológico relevante. **(** ***Modificado*** **)**
    **(III)** **Hiperatividade detrusora não neurogênica** **(secundária):** como observado no **item 3.4.5.2.3,** existe causa não neurológica identificável possível para as contrações involuntárias do detrusor durante o enchimento vesical. Por exemplo: funcional (obstrução), cálculo, tumor (por exemplo, carcinoma *in situ* ) e ITU. **(** ***Modificado*** **)**
  **5.1.3** **Disfunção de armazenamento e complacência** **reduzida:** esse diagnóstico por sintomas e investigações urodinâmicas é feito em indivíduos com sintomas do TUI, mais comumente sintomas de armazenamento, quando há aumento não fásico (às vezes linear ou exponencial) na pressão detrusora durante a cistometria com capacidade geralmente reduzida, indicando complacência reduzida **(item 3.4.6).** **(** ***Novo*** **)**
    **5.1.3.1 Incontinência e complacência reduzida:** incontinência urinária diretamente relacionada à DACR. **(** ***Novo*** **)**
**5.2 Fator infravesical** (disfunção da uretra/esfíncter, diminuição da resistência uretral e incompetência/insuficiência)
  **5.2.1** Incontinência de esforço urodinâmica _R__5.7_
    **5.2.1.1 Definição:** conforme observado no **item 3.6.2.1,** esse diagnóstico clínico por investigação de ***sintomas, sinais e urodinâmica*** envolve o achado de perda involuntária durante a cistometria, associado a aumento da pressão intra-abdominal, na ausência de contração do músculo detrusor. _NR 5.7-NR 5.10_
    **5.2.1.2 Subtipo:** **deficiência esfincteriana intrínseca** **(item 3.6.2.1.1):** mecanismo de fechamento uretral muito enfraquecido. **(** ***Modificado*** **)**
    **DISFUNÇÃO MICCIONAL:** Os diagnósticos relacionados ao esvaziamento da bexiga anormalmente lento e/ou incompleto manifestam-se como fluxo de urina anormalmente lento **(item 3.1.10)** e/ou RPM anormalmente alto **(item 3.2.2),** com confirmação por estudo pressão/fluxo, incluindo qualquer imagem relacionada. **(** ***Novo - masculino*** **)**
**5.3 Fator vesical (atividade detrusora fraca ou ausente)**
  **5.3.1 Hipoatividade do detrusor (HAD)**_NR 5.11_
    **5.3.1.1 Definição de HAD:** conforme o **item 3.7.3.2,** o diagnóstico é feito com base na ***investigação urodinâmica*** , geralmente (mas nem sempre) com ***sintomas e sinais*** relevantes manifestados por baixa pressão detrusora ou curta contração detrusora em combinação com baixo fluxo urinário **(item** **3.1.10),** resultando em esvaziamento prolongado da bexiga e/ou falha em atingir o esvaziamento completo da bexiga dentro de um período de tempo normal, com ou sem um alto RPM **(item** **3.2.2)** (conforme “detrusor hipocontrátil”: contração detrusora de força reduzida). **(** ***Modificado*** **)**
  **5.3.2 A contratilidade detrusora**_NR 5.11_
    **5.3.2.1 Definição de contratilidade detrusora:** conforme **item 3.7.3.3** . Diagnóstico por **investigação urodinâmica** , geralmente (mas nem sempre) com **sintomas e sinais** relevantes manifestados pela ausência de contração detrusora observada durante o estudo miccional, resultando em esvaziamento prolongado da bexiga e/ou falha em atingir o esvaziamento completo da bexiga dentro de um período normal. A micção em homens com contratilidade detrusora é geralmente obtida por esforço ou pressão manual na bexiga, resultando, em geral, em uma taxa de fluxo urinário anormalmente lento **(item 3.1.10** ) e/ou um RPM anormalmente alto **(item 3.2.2) (** ***Modificado*** **)**
    **5.3.2.2 Subtipos:**
    - Contratilidade neurogênica **(item 3.7.3.3.1).**
    - Contratilidade detrusora não neurogênica **(item 3.7.3.3.2).**
**5.4 Fator infravesical (disfunção uretral/esfíncter)**
  **5.4.1 Obstrução infravesical**_NR 5.1, NR 5.12_
    **5.4.1.1 Definição de OIV:** o diagnóstico é feito com base em ***investigação urodinâmica (estudo pressão/fluxo imagens)*** , geralmente (mas não sempre) com ***sintomas*** e/ou sinais relevantes, manifestados por fluxo urinário anormalmente lento **(item 3.1.10)** , _NR 5.12_ com evidência de pressão detrusora de micção anormalmente alta e fluxo de urina anormalmente lento **(item 3.8.2.1)** durante o estudo miccional com ou sem RPM anormalmente alto **(item 3.2.2)** . _NR 5.13_ **(** ***Modificado*** **)**
    **5.4.1.2** **Possíveis locais/causas de OIV podem** **ser:**
      **5.4.1.2.1 Funcional:** obstrução do colo vesical, disfunções vesicoesfincterianas, hiperatividade do AP. **(** ***Novo*** **)**
      **5.4.1.2.2** **Mecânica:** aumento prostático benigno, estenose uretral, estenose meatal. _NR 5.14-NR 5.19_
      Exames de imagem do TUI, especialmente a videourodinâmica e a EMG, podem ser necessários para avaliar a localização/causa. **(** ***Novo*** **)**
  **5.4.2 Apresentações alternativas da disfunção miccional**
    **5.4.2.1 Retenção urinária aguda:** o indivíduo é incapaz de urinar apesar de ter uma bexiga cheia, que, ao exame, é dolorosamente distendida e facilmente palpável e/ou percutível. **(** ***Mo*** ***dificado*** **)**
    **5.4.2.2 Retenção urinária crônica:** geralmente (mas nem sempre) a bexiga é indolor e palpável ou percutível, com RPM crônico alto. O paciente apresenta fluxo lento e esvaziamento vesical incompleto crônico, mas pode ser assintomático. Pode ocorrer incontinência por transbordamento. Alguns homens com retenção apresentam comprometimento da função renal e/ou hidronefrose. **(** ***Modificado*** **)**
    **5.4.2.3 Agudização na retenção crônica:** um indivíduo com retenção crônica entra em retenção aguda e é incapaz de urinar. **(** ***Novo*** **)**
    **5.4.2.4 Retenção com transbordamento:** perda involuntária de urina diretamente relacionada a uma bexiga excessivamente cheia em retenção. **(** ***Novo*** **)**
**5** **.5 Disfunção de armazenamento e esvaziamento mista**
  **5.5.1 Obstrução infravesical e hipoatividade do detrusor**
    **5.5.1.1 Definição:** OIV urodinâmica **(item 3.8.2.1),** que ocorre em sincronia com a HAD urodinâmica **(item 3.7.3.2)** no estudo pressão fluxo _NR__5.20_ **(** ***Novo*** **)**
  **5.5.2 Hiperatividade detrusora e OIV**_NR 5.1_
    **5.5.2.1 Definição:** hiperatividade detrusora urodinâmica **(item 3.4.5.2)** na cistometria de enchimento na presença de OIV **(item 3.8.2.1)** no estudo fluxo/pressão. _NR 5.21_ **(** ***Novo*** **)**
  **5.5.3** Hiperatividade detrusora com HAD
    **5.5.3.1 Definição:** hiperatividade detrusora urodinâmica **(item 3.4.5.2)** na cistometria de enchimento em combinação com HAD urodinâmica **(item 3.7.3.2).** No estudo pressão/fluxo, o diagnóstico destina-se a substituir a antiga expressão “hiperatividade detrusora com contratilidade prejudicada” e sua sigla, HDCP. É mais comum no grupo de idosos. **(** ***Novo*** **)**

## Notas de rodapé para da seção 5

5.1: Dados de grandes séries sobre a frequência relativa de diagnósticos em homens que apresentam sintomas de disfunção de TUI/AP são escassos. A prevalência relativa de seis diagnósticos principais é conhecida em mulheres. ^( 4 , 5 )^ Em uma série de 504 homens consecutivos ^( 64 )^ com 49 a 94 anos, encaminhados para estudo urodinâmico, incluindo videouretrocistografia e revisão departamental de resultados devido a sintomas urológicos. Os seguintes diagnósticos foram feitos:

**Hiperatividade do detrusor (HD)** em 149 (29,6%)

deles, HD mais obstrução (OIV) em 124 (24,6%) –

ou seja, HD **total de (54,2%)**

Obstrução (OIV) isolada em 161 (31,9%)

ou seja, OIV **total de 56,5%**

diagnóstico normal/não específico em 70 (13,9%).

Alguns diagnósticos mais recentes podem não ter existido em 1990.

5.2: Prevalência de hipersensibilidade vesical: no estudo EPIC, ^( 115 )^ a taxa de prevalência para homens que urinam com frequência superior a oito vezes ao dia é de aproximadamente 12%. A presença de hipersensibilidade vesical em pacientes uroginecológicas é de 10% a 13%. ^( 5 )^

5.3: Não deve haver ITU conhecida ou suspeita. A hipersensibilidade vesical é, muitas vezes, um diagnóstico de exclusão, após outras doenças mais sérias, como malignidade do TUI, incluindo o carcinoma *in situ* da bexiga, serem excluídas.

5.4: Prevalência de incontinência urinária total em homens por idade: ^( 116 )^ 19 a 44 anos: 4,8%; 45 a 64 anos: 11,2%; 65 a 79 anos: 21,1% e >80 anos: 32,2%.

5.5: Prevalência de incontinência de urgência (urinária) em homens por idade: ^( 117 )^ 19 a 44 anos: 3,1%; 45 a 64 anos: 7,8%; 65 a 79 anos: 11,7% e >80: 18,1%.

5.6: As contrações anormais do detrusor podem ser, às vezes, observadas durante a cistometria, sem que o paciente seja sintomático.

5.7: Prevalência de incontinência de esforço urodinâmica: prevalência de incontinência de esforço (urinária) em homens por idade: ^( 115 )^ 19 a 44 anos: 0,7%; 45 a 64 anos: 3,8%; 65 a 79 anos: 2,7%; >80 anos: N/A – ou no total para homens acima de 18 anos ^( 117 )^ (1,4%).

5.8: Os homens, diferentemente das mulheres, não desenvolvem hipermobilidade uretral significativa (com a possível exceção da prostatectomia radical) e, portanto, a incontinência urodinâmica é mais frequentemente associada à deficiência intrínseca do esfíncter do que à hipermobilidade uretral. A deficiência esfincteriana é mais comumente resultante de trauma pélvico ou pós-prostatectomia, seja transuretral ou radical, ou distúrbio neurológico.

5.9: A prevalência de incontinência urinária pós-ressecção transuretral da próstata para doença prostática benigna ocorre entre 0,5% e 3%. ^( 118 - 122 )^

5.10: A prevalência para pós-prostatectomia radical: as taxas de incontinência pós-prostatectomia radical variam dependendo da definição utilizada e da duração do acompanhamento. No entanto, a incidência a longo prazo varia entre 4% e 8%. ^( 117 - 122 )^

5.11: A prevalência de HAD ou de contratilidade detrusora: em estudo envolvendo uma revisão de dados urodinâmicos de 1.179 pacientes com 65 anos ou mais, Jeong et al. relataram a prevalência de HAD de 40,2% em homens. ^( 123 )^

5.12: A OIV urodinâmica pode ser diagnosticada usando o nomograma da ICS. ^( 89 )^ A fórmula usada, conhecida como índice de IOIV é calculada pela P _det_ .Q _max_ menos duas vezes o fluxo urinário máximo (IOIV = P _det_ .Q _max_ – 2 Q _max_ ). Um IOIV com valor >40 define OIV, menos de 20 define ausência de OIV e, entre esses valores, OIV incerta. Classificações alternativas para OIV são as de Schäfer (zero a VI) ^( 90 , 91 )^ e CHESS. ^( 92 )^

5.13 As evidências em homens sobre RPM e OIV não são claras. Estudo urodinâmico em doentes adultos do sexo masculino com HBP clínica demonstraram que aproximadamente 30% dos homens com RPM ≥50mL não têm OIV/OPB, independentemente da magnitude do RPM, ^( 124 )^ e, da mesma forma, 24% dos homens com OIV/OPB urodinamicamente confirmadas têm RPM <50mL ou mesmo 0mL. ^( 124 , 125 )^

5.14: O nível de obstrução geralmente pode ser diagnosticado durante a videourodinâmica com uretrocistografia miccional. Pode ser auxiliado por EMG do esfíncter ou do AP durante a micção.

5.15: OIV decorrente de um aumento da próstata: OIV, cuja causa é o aumento benigno da próstata, com evidência clínica ou de imagem.

5.16: OIV do colo vesical: OIV cuja causa está no nível do colo vesical (clínica ou radiológica). O traçado eletromiográfico do AP (EMG) deve ser silencioso durante a micção nesses pacientes.

5.17: OIV devido à hiperatividade muscular do AP: OIV cuja causa está no nível da MAP (clínica, urodinâmica ou radiológica). O traçado da EMG do AP pode não ser positivo durante a micção.

5.18: OIV do rabdoesfíncter (esfíncter urinário externo): OIV cuja causa é ao nível de rabdoesfíncter (clínico, urodinâmico ou radiológico). O traçado eletromiográfico do AP (EMG) pode não ser positivo durante a micção.

5.19: OIV devido à estenose do colo vesical ou da uretra devido à fibrose: a estenose do colo vesical pode ocorrer secundariamente à cirurgia da próstata, por conta de doença benigna, cirurgia radical da próstata, radioterapia ou trauma.

5.20: Atualmente, embora muitos especialistas nesse campo concordem que essa entidade exista, não há consenso sobre sua definição, dada a indefinição da HAD. Existe o nomograma de Maastricht-Hannover, ^( 126 )^ que pode ser usado para diagnosticar contratilidade detrusora reduzida na presença de obstrução (ou vice-versa).

5.21: Até 83% ^( 127 )^ dos homens com OIV urodinâmica podem ter HD urodinâmico concomitante. Ambos o grau de OIV e a idade avançada foram fatores independentes de HD em homens. Quanto mais grave a OIV, maior a chance de HD.

## ÁREAS PARA PESQUISAS FUTURAS

Na preparação deste documento, as seguintes lacunas no conhecimento da disfunção masculina do TUI/AP foram notadas comparadas com o equivalente sobre disfunção de TUI/AP feminina: ^( 5 )^

- RPM em homens com sintomas de disfunção de TUI/AP.
- Dados da diurese masculina.
- Complacência vesical (valores normais e anormais nos homens).
- Grandes séries adicionais de pacientes para os dados de prevalência e a frequência relativa dos diagnósticos masculinos mais comuns. ^( 64 )^
